# Methyltransferase-directed orthogonal tagging and sequencing of miRNAs and bacterial small RNAs

**DOI:** 10.1186/s12915-021-01053-w

**Published:** 2021-06-22

**Authors:** Milda Mickutė, Kotryna Kvederavičiūtė, Aleksandr Osipenko, Raminta Mineikaitė, Saulius Klimašauskas, Giedrius Vilkaitis

**Affiliations:** Institute of Biotechnology, Life Sciences Center, Vilnius University, LT-10257 Vilnius, Lithuania

**Keywords:** Methyltransferase, RNA modification, RNA-seq, Non-coding RNA, Epitranscriptome, Probiotic

## Abstract

**Background:**

Targeted installation of designer chemical moieties on biopolymers provides an orthogonal means for their visualisation, manipulation and sequence analysis. Although high-throughput RNA sequencing is a widely used method for transcriptome analysis, certain steps, such as 3′ adapter ligation in strand-specific RNA sequencing, remain challenging due to structure- and sequence-related biases introduced by RNA ligases, leading to misrepresentation of particular RNA species. Here, we remedy this limitation by adapting two RNA 2′-O-methyltransferases from the Hen1 family for orthogonal chemo-enzymatic click tethering of a 3′ sequencing adapter that supports cDNA production by reverse transcription of the tagged RNA.

**Results:**

We showed that the ssRNA-specific DmHen1 and dsRNA-specific AtHEN1 can be used to efficiently append an oligonucleotide adapter to the 3′ end of target RNA for sequencing library preparation. Using this new chemo-enzymatic approach, we identified miRNAs and prokaryotic small non-coding sRNAs in probiotic *Lactobacillus casei* BL23. We found that compared to a reference conventional RNA library preparation, methyltransferase-Directed Orthogonal Tagging and RNA sequencing, mDOT-seq, avoids misdetection of unspecific highly-structured RNA species, thus providing better accuracy in identifying the groups of transcripts analysed. Our results suggest that mDOT-seq has the potential to advance analysis of eukaryotic and prokaryotic ssRNAs.

**Conclusions:**

Our findings provide a valuable resource for studies of the RNA-centred regulatory networks in *Lactobacilli* and pave the way to developing novel transcriptome and epitranscriptome profiling approaches in vitro and inside living cells. As RNA methyltransferases share the structure of the AdoMet-binding domain and several specific cofactor binding features, the basic principles of our approach could be easily translated to other AdoMet-dependent enzymes for the development of modification-specific RNA-seq techniques.

**Supplementary Information:**

The online version contains supplementary material available at 10.1186/s12915-021-01053-w.

## Background

Designer chemical moieties installed in a targeted and selective manner on biopolymers can serve as orthogonal handles for their visualisation, manipulation and sequence analysis. As enzymes are the most selective and efficient catalysts known, chemo-enzymatic strategies based on repurposing transferase reactions to accept chemically modified transferrable groups are gaining an increased popularity. By far, the most widely used are *S*-adenosyl-L-methionine- (AdoMet-) dependent methyltransferases, which offer a broad range of tagging chemistries that can be deposited in vitro and in vivo [1, 2]. RNA methyltransferases are the most abundant class of RNA modifying enzymes. They incorporate a methyl group into transfer RNAs (tRNAs), ribosomal RNAs (rRNAs), messenger RNAs (mRNAs), long non-coding RNAs (lncRNAs) and various small non-coding RNAs (ncRNAs), including small nuclear RNAs (snRNAs), small nucleolar RNAs (snoRNAs), microRNAs (miRNAs) and their precursors, small interfering RNAs (siRNAs) or Piwi-interacting RNAs (piRNAs) [3]. Some of these enzymes have been successfully exploited to develop novel RNA labelling and mapping approaches [1] and techniques for identifying natural modification sites and positions [4, 5].

Members of the Hen1 2′-O-methyltransferase subfamily catalyse the transfer of a methyl group from *S*-adenosyl-L-methionine onto the 2′-O-ribose of the 3′ terminal nucleotide to protect RNA from degradation [6]. A particular Hen1 enzyme typically modifies only a definite type or a subset of RNA substrates. For example, the plant HEN1 from *Arabidopsis thaliana* (AtHEN1) preferentially methylates 21-24 bp long double-stranded miRNAs and siRNAs [7, 8], while the animal DmHen1 from *Drosophila melanogaster* is a short single-stranded piRNA and siRNA specific methyltransferase [9]. However, it has been shown that DmHen1 and especially its isolated catalytic domain DmHen1ΔC can also efficiently modify ssRNA molecules up to 80 nt at least in vitro [10]. AtHEN1 methylation-dependent chemoselective small RNA cloning combined with next-generation sequencing has enabled the cell-type-specific miRNAs profiling in complex animal tissues even using the endogenous AdoMet cofactor [11]. Recently, Hen1 enzymes have been repurposed for the deposition of user-defined functional or reporter groups, such as fluorophore or biotin, onto the 3′ end of the RNA strand [10, 12, 13].

In this work, we explored if such orthologous linking chemistry can be applied to advance the profiling of cellular small RNA pools. Existing RNA-seq technologies involve enzymatic ligation of sequencing adapters using RNA ligases, which are known to suffer from structure and sequence related biases leading to misrepresentation of particular RNA species [14–18]. Here, we used the DmHen1 and AtHEN1 RNA methyltransferases followed by chemical click ligation to specifically tether adapters to the 3′ ends of single-stranded RNAs or double-stranded miRNAs and siRNAs, respectively. Remarkably, we found that certain reverse transcriptases were capable of faithfully producing cDNA from selected orthogonally tethered adapters. The accuracy of the newly developed methyltransferase-Directed Orthogonal Tagging and RNA sequencing (mDOT-seq) method was evaluated by using the gold standard miRXplore Universal Reference RNA. Finally, we successfully used this approach to characterise a small non-coding RNA (sRNA) transcriptome of *Lactobacillus casei*, which is one of the most exploited probiotic bacteria from the lactic acid bacteria (LAB) group.

## Results

### Hen1 2′-O-methyltransferase-directed tagging of ssRNAs and short dsRNAs for selective cDNA production

**Fig. 1:**
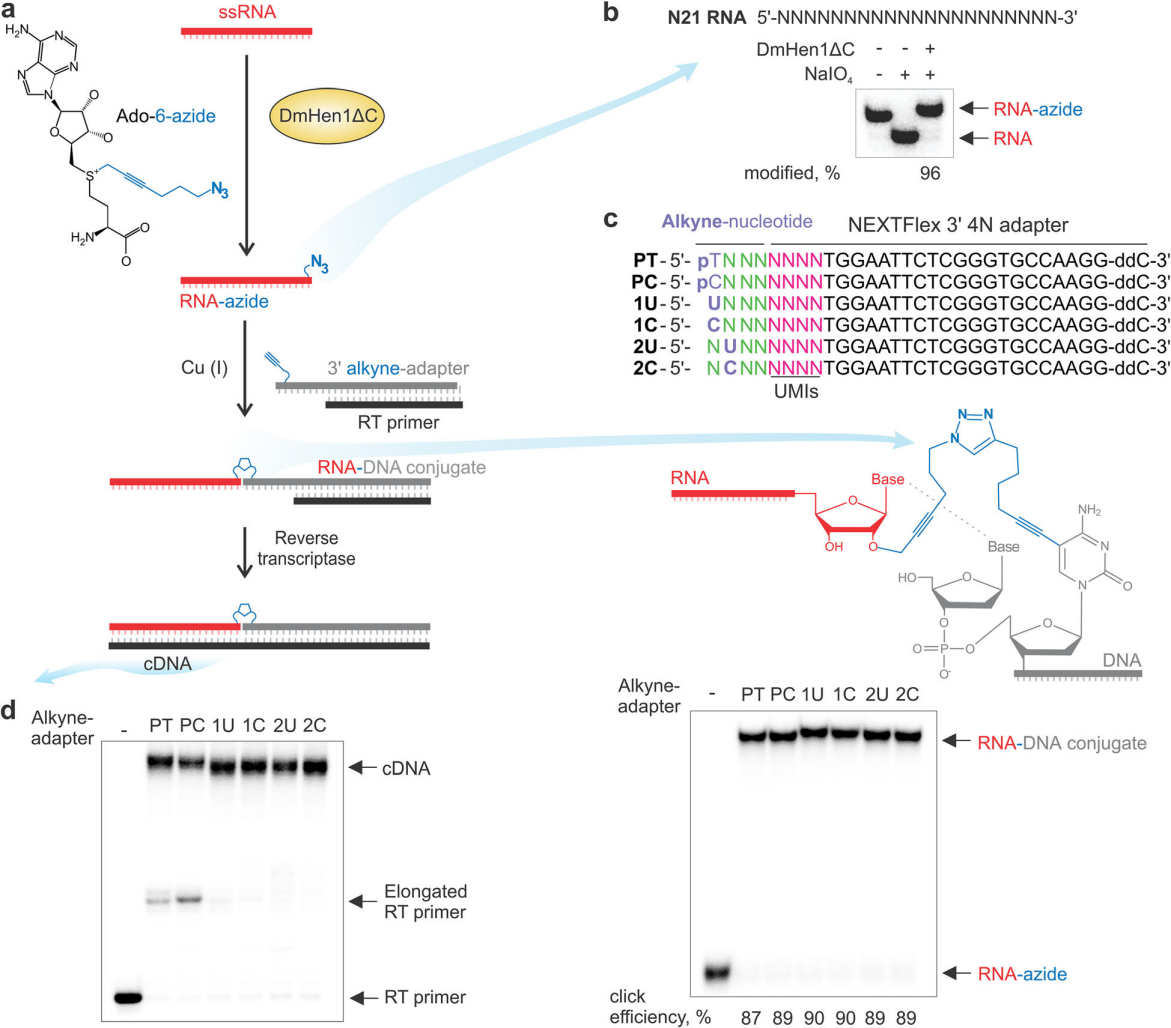
Selective DmHen1ΔC methyltransferase-directed attachment of a sequencing adapter to the 3′ end of single-stranded RNA. **a** A principal scheme of bioorthogonal attachment of 3′ alkyne-adapter/RT primer for cDNA synthesis. ssRNA is functionalized with a hexynyl-azide group by the action of the DmHen1ΔC methyltransferase in the presence of the cofactor analogue Ado-6-azide. The resulting RNA-azide is conjugated to a 3′ alkyne-adapter/RT primer in the presence of Cu(I) yielding a covalent RNA-DNA conjugate which serves as a template for the cDNA synthesis. **b** DmHen1ΔC-directed azide-tagging of a random pool of 21-mer ssRNAs. Reactions were performed with 2 μM of DmHen1ΔC, 0.2 μM 5′-^32^P-labelled N21 RNA and 0.1 mM Ado-6-azide. The fraction of modified RNA was estimated after sodium periodate-mediated oxidation/β-elimination of the 3′ terminal nucleotide of unmodified RNA as described in [19]. **c** Chemical conjugation of RNA-azide with a 3′ alkyne-adapter. Reactions were carried out in 55% DMSO using 0.2 μM ^32^P-RNA-azide, 10 μM 3′ alkyne-adapter/RT primer and 3.3 mM CuBr-TBTA. Outlined are the 5′ terminal sequences of the 3′ adapters with different alkyne moieties tested in this study. **d** Reverse transcription through different conjugation linkers. RNA-DNA conjugates were prepared in click reactions containing 20 μM of RNA-azide and 5 μM of 3′ alkyne-adapter/^32^P-RT primer. Each RNA-DNA conjugate (10 nM) was reverse transcribed in reaction containing 10 U/μl of RevertAid Reverse Transcriptase and 0.25 mM dNTP

We have previously demonstrated that AtHEN1 and an engineered version of the DmHen1 methyltransferase, DmHen1ΔC, can transfer six-carbon linear chains carrying terminal amine or azide functional groups from synthetic analogues of the *S*-adenosyl-L-methionine (AdoMet) onto the ribose of the 3′ terminal nucleotides [10, 12, 13]. While AtHEN1 is specific for RNA duplexes of a defined length, DmHen1ΔC modifies ssRNA substrates with no apparent size limitation. To explore if these reactions can be repurposed for orthogonal covalent conjugation of sequencing adapters to the 3′ ends of corresponding RNA species (the core steps of which are depicted in Fig. 1a), we started with a pool of synthetic randomised N21 RNA oligonucleotides containing 4.4 × 10^12^ RNA sequence variants. We found that DmHEN1ΔC-directed 3′ terminal modification of this RNA pool using the Ado-6-azide cofactor occurred to near completion, demonstrating that the six-carbon propargylic linker with a terminal azide group can be efficiently attached to random sequences (Fig. 1b). In the next chemical ligation step, we used an alkyne-modified adapter suitable for the reverse transcription reaction. To minimise the interference of the linker in subsequent enzymatic steps, our choice fell on a copper (I)-catalysed azide-alkyne cycloaddition (CuAAC) reaction, because it produces the least bulky and least rigid cycloaddition product of defined stereochemistry (Fig. 1c illustrates a linear chain with a central triazole ring) as opposed to copper-free strain-promoted reactions which typically rely on bulky cyclooctyne derivatives (such as DBCO, BCN, DIFO) [20]. The efficiency of the click reaction was assessed using six different synthetic DNA adapters with extended alkyne moieties tethered to the 5′ terminal phosphate groups or to the C5 position of the first or second 5′ terminal cytosine/uracil residues (Additional file 1: Fig. S1). These tethering chemistries were chosen based on synthetic availability and structural considerations (persistence length and conformational flexibility of the covalent tether required to promote the base stacking between the tagged 3′ terminal nucleotide of the RNA strand and the 5′ terminal nucleotide of the attached adapter) [21]. Excellent yields of RNA-DNA conjugates were achieved with all six adapter types (Fig. 1c) even at low-nanomolar concentrations of azide-functionalised RNAs (Additional file 1: Fig. S2). Finally, the obtained six conjugates were then examined for their suitability to support the reverse transcription reaction. Remarkably, initial strand extension experiments performed with RevertAid Reverse Transcriptase showed a predominant formation of expected full-length cDNA products, as exemplified in Fig. 1d. Minor amounts of shorter cDNA fragments (where RT primer was extended up to covalent linker) accumulated using both RNA-DNA conjugates with alkyne-modified 5′ phosphate bearing adapters PT and PC indicating that this type of linker is less well tolerated by the enzyme. In contrast, no partially extended products were observed using templates with alkyne at the C5 position of the pyrimidine nucleobase, namely the 1C, 1U, 2C and 2U adapters. cDNA synthesis performed with M-MuLV reverse transcriptase and four of its mutants revealed high yields of the full-length product (Additional file 1: Fig. S3). However, variants lacking RNase H activity displayed an improved ability to traverse the orthogonal linkers. Most of the RevertAid transcripts terminating on the opposite side of the linker were appended with a short 3′ tail (1–3 extra nucleotides), apparently due to the stronger template-independent nucleotidyl-transferase activity or protein dissociation after the synthesis of only a few nucleotides following DNA-RNA junction [22].

**Fig. 2:**
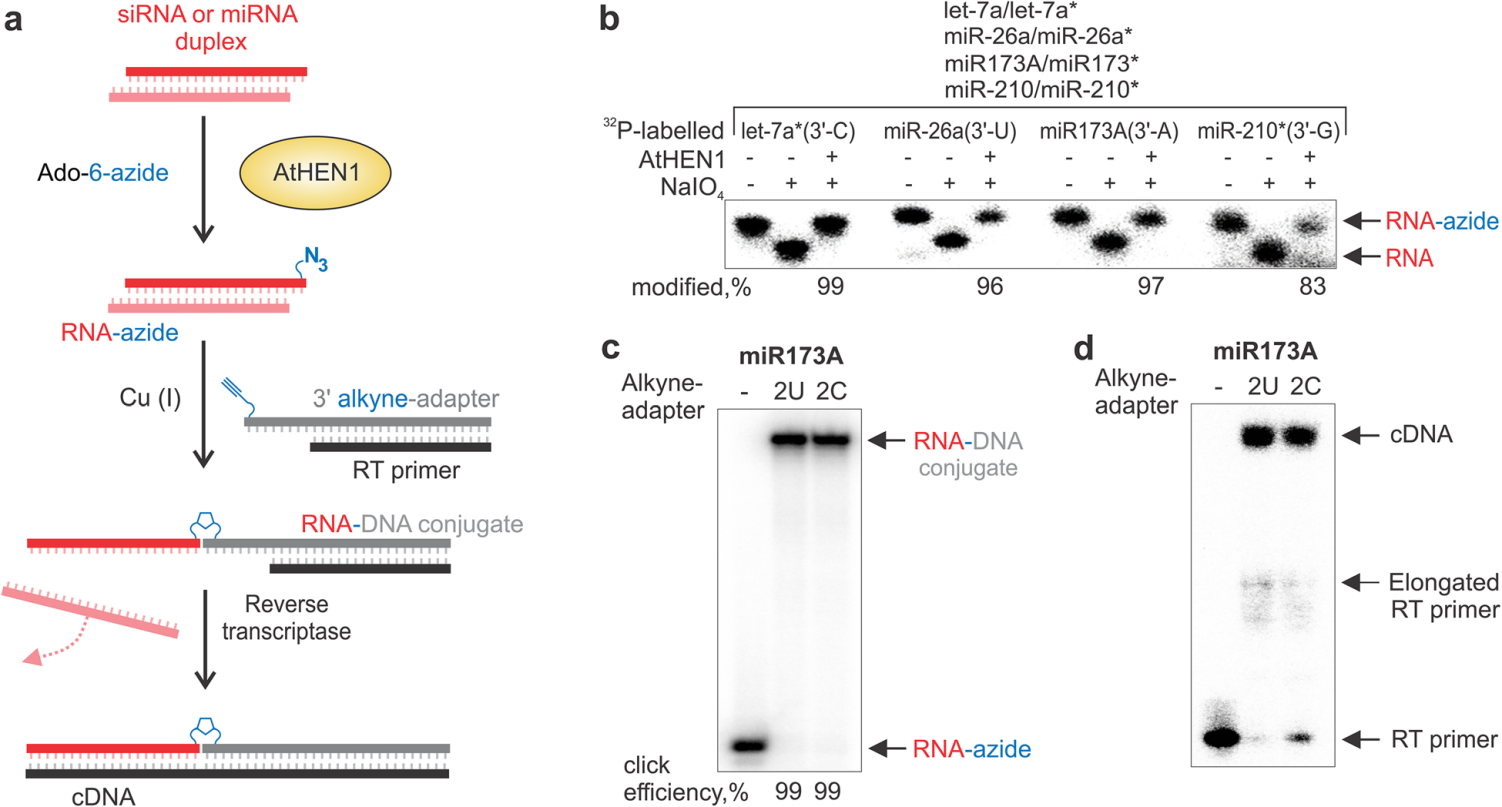
AtHEN1 methyltransferase-directed attachment of a sequencing adapter to the 3′ end of small non-coding RNA duplexes. **a** An overview of AtHEN1 application. **b** AtHEN1-directed azide-tagging of substrate RNAs with different 3′ terminal nucleotides. 2 μM of methyltransferase and 400 μM Ado-6-azide were incubated with the mixture of four miRNA/miRNA* duplexes (0.05 μM of each) in which particular guide or passenger strand was ^32^P-labelled (let-7a*, miR-26a, miR173A or miR-210* with C, U, A or G at the 3′ end, respectively). After the treatment with sodium periodate samples were analysed by denaturing PAGE. **c** ^32^P-miR173A/miR173* is efficiently attached to 2U and 2C alkyne-adapter/RT primer. Click reaction was carried out under the same conditions as described in Fig. 1c. **d** cDNA synthesis from miR173A coupled with 2U or 2C alkyne-adapter. 10 nM of RNA-DNA conjugate were reverse transcribed using ^32^P-RT primer as described in Fig. 1d

Analogous experiments were carried out with the AtHEN1 enzyme, which selectively modifies short double-stranded RNAs containing dinucleotide 3′ overhangs, such as miRNAs and siRNAs (Fig. 2a) [23–25]. Using the Ado-6-azide cofactor, we observed efficient functionalization of both natural miRNA/miRNA* (guide/passenger strand*) duplexes and also of individual miRNA strands in heteroduplexes with addressing DNA oligonucleotide probes, miRNA/DNA (Additional file 1: Fig. S4) [13]. Similarly, the modification reaction using a mixture of four miRNA/miRNA* duplexes confirmed efficient enzymatic derivatization of all tested guide miRNAs, each containing a different 3′ terminal nucleotide (Fig. 2b). Following the click-conjugation of the alkyne-DNA adapter to the 3′ terminally azide-modified RNA (Fig. 2c), the miRNA template was efficiently converted into full-length cDNA via reverse transcription (Fig. 2d).

Thus, we showed that both Hen1 methyltransferases can efficiently append RNA pools with a 3′ terminal azide functionality, which, in turn, can serve as an orthogonal handle for covalent tethering of a DNA adapter suitable for generating cDNA via enzymatic reverse transcription reaction.

### Optimization and validation of the mDOT-seq approach with a reference set of miRNAs

**Fig. 3:**
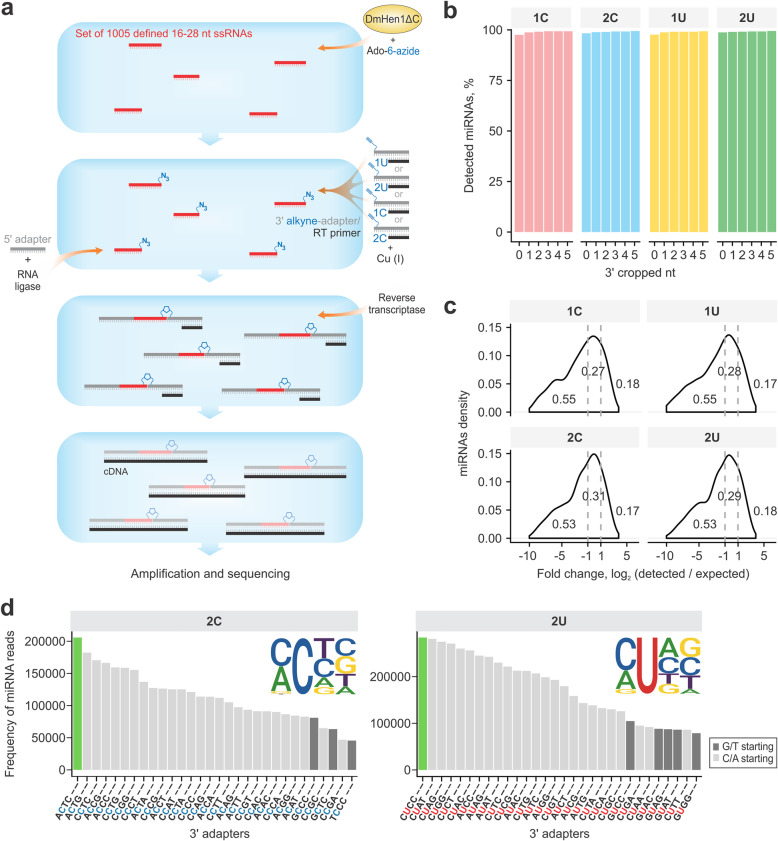
Selection of alkyne-adapters through sequencing of miRXplore Universal Reference RNA libraries. **a** A schematic of the mDOT-seq. **b** The majority of miRNAs are detected as full-length sequences in all libraries. Bar plot shows the observed fraction of miRNAs after increasing number of nucleotides is cropped from its 3′ end. The exact numbers are provided in Additional file 1: Table S1. **c** 2C and 2U libraries contain the highest portion of accurately quantified miRNA reads. miRNAs identified in the reference set of sequences cropped of two nt at their 3′ end and having at least 1 CPM in each library were used to calculate fold-deviation from the equimolar input and plotted as log_2_ values. Values within two folds from the expected ones (grey vertical lines) were considered unbiased according to [27]. **d** Top 30 of 2C or 2U 3′ adapters containing different bases at first, third and fourth 5′ positions with the highest number of identified reads. The libraries were demultiplexed using each of 3′ adapters and the total number of miRNA reads was calculated. Sequence logos of top ranked adapters were created using WEBLOGO [28]. The data underlying the presented graphs are in Additional file 2

To assess the capacity of the DmHEN1ΔC-mediated sequencing technology, named mDOT-seq (methyltransferase-Directed Orthogonal Tagging and RNA sequencing), for high-throughput analysis of short RNAs, we exploited the gold standard miRXplore Universal Reference. This RNA pool consists of 1005 mature human, mouse, rat, and viral miRNAs ranging between 16 and 28 nt in length, with individual oligoribonucleotides present in equimolar concentrations, which thus permits a comprehensive calibration of analytical techniques that aim at tackling the complexity of cellular miRNAome [26]. We constructed four variants of cDNA libraries employing the previously selected 1C, 1U, 2C and 2U alkyne-adapters (the NEXTflex Small RNA-seq Kit v3 protocol was applied starting from 5′ adapter ligation step) and subjected them to Illumina sequencing (Fig. 3a). Since experimental replicates (two sequencing libraries prepared separately for each 3′ adapter) were nearly identical (Additional file 1: Fig. S5a), their reads were pooled for further analysis. Pairwise comparisons revealed the strongest correlation between the 2C and 2U libraries, indicating that moderately divergent RNA pools were captured using the 1C and 1U 3′ adapters (Additional file 1: Fig. S5b). Moreover, the 2C and 2U preparations detected slightly higher amounts of full-length miRNAs: 98.3–98.7% in 2C, 2U versus 97.5% in 1C and 1U libraries (Fig. 3b and Additional file 1: Table S1). This difference became more apparent at higher detection thresholds (Additional file 1: Fig. S5c). Since the mapping of reads to the miRXplore sequences after trimming of 3′ terminal nucleotides increased the number of miRNA species identified (Fig. 3b and Additional file 1: Table S1), we suggest that the secondary structures of the 1C and 1U 3′ adapters may impact the precision of *bona fide* 3′ end calling to a greater extent than that of the 2C and 2U 3′ adapters. Also, the abundance of different RNAs in the 2C and 2U libraries showed a smaller deviation from uniformity than in the 1C and 1U libraries (Fig. 3c). Altogether, our results revealed that the 3′ adapters with alkyne moieties attached to the fifth carbon atom of the second cytosine/uracil are best suited for the preparation of RNA sequencing libraries.

To understand the representation bias in the sequencing libraries, we analysed miRNAs comprising 10% of high-, medium- and low-abundant species as well as undetected ones. As shown in Additional file 1: Fig. S6, RNAs prone to forming double-stranded structures or having only short 3′ overhangs were underrepresented in all libraries. This appears to be consistent with the requirement of a single-stranded 3′ end for DmHen1 activity [10]. Other characteristics of the RNA sequences did not contribute to the RNA detection inequalities, whereas guanine-rich RNAs with higher Tm tended to be overrepresented. A detailed analysis of the preferred/disfavoured bases at 1–16 positions upstream of the 3′ end revealed predisposition of detected RNAs to be repleted with G but depleted of T, A and C nucleotides from some individual sites and lower representation of RNAs containing adenine at the 3′ position (Additional file 1: Fig. S7). Since the enzymatic examination revealed 3′ terminal adenines as highly desirable targets for DmHen1ΔC [10], we suggest that uneven representativeness reflects the reverse transcription or cDNA amplification biases. Thus, further optimization of the reaction conditions or selection of an RT enzyme could further reduce the inequality in the sequenced transcripts’ population.

Finally, to select the optimal flanking sequences surrounding the 2C-alkyne and 2U-alkyne nucleotides in the 3′ adapters, we calculated the transcript counts for each particular adapter sequence varying at the first, third and fourth positions (Fig. 3d). This analysis revealed a strong A/C enrichment at the 5′ end but a weak nucleotide-dependence at the 3rd and 4th positions (except for a disfavour for 4th A), suggesting that the sequence preference was mostly caused by the first nucleotide. Among the top-ranked adapters, DNA oligonucleotides containing ACTC and CUCC motifs at the 5′ end accumulated the highest number of reads and gave the most accurate quantification of miRNAs (Fig. 3d and Additional file 1: Fig. S8) [27]. Therefore, we suggest that these sequences of 3′ alkyne-adapters are optimal for effective sequencing and can be used in further experiments.

Taken together, these results show that the developed mDOT-seq procedure is capable of identifying a broad range of RNA targets and thus can be exploited for various miRNA applications. Inspired by this finding, we went on to examine the capacity of mDOT-seq for profiling of cellular single-stranded RNAs.

### The discovery of small non-coding RNAs in *Lactobacillus casei* using mDOT-seq

To evaluate if mDOT-seq can be applied for RNA profiling in biological organisms, we went on to characterise small non-coding RNAs (sRNAs) from *Lactobacillus casei* strain BL23 (NCBI changed the organism’s name to *Lactobacillus paracasei* BL23 in August 2020). It is an important model organism for understanding fundamental aspects of cell biology of lactic acids bacteria (LAB) which are widespread in the human microbiome and prominent components of probiotic compositions [29–31]. Like 50–500 nt sRNAs from well-characterised bacteria [32, 33], sRNAs of LAB could potentially play essential regulatory roles in shaping different physiological processes, cellular response to biotic or abiotic stresses as well as adaptation in the gastrointestinal tract.

**Fig. 4:**
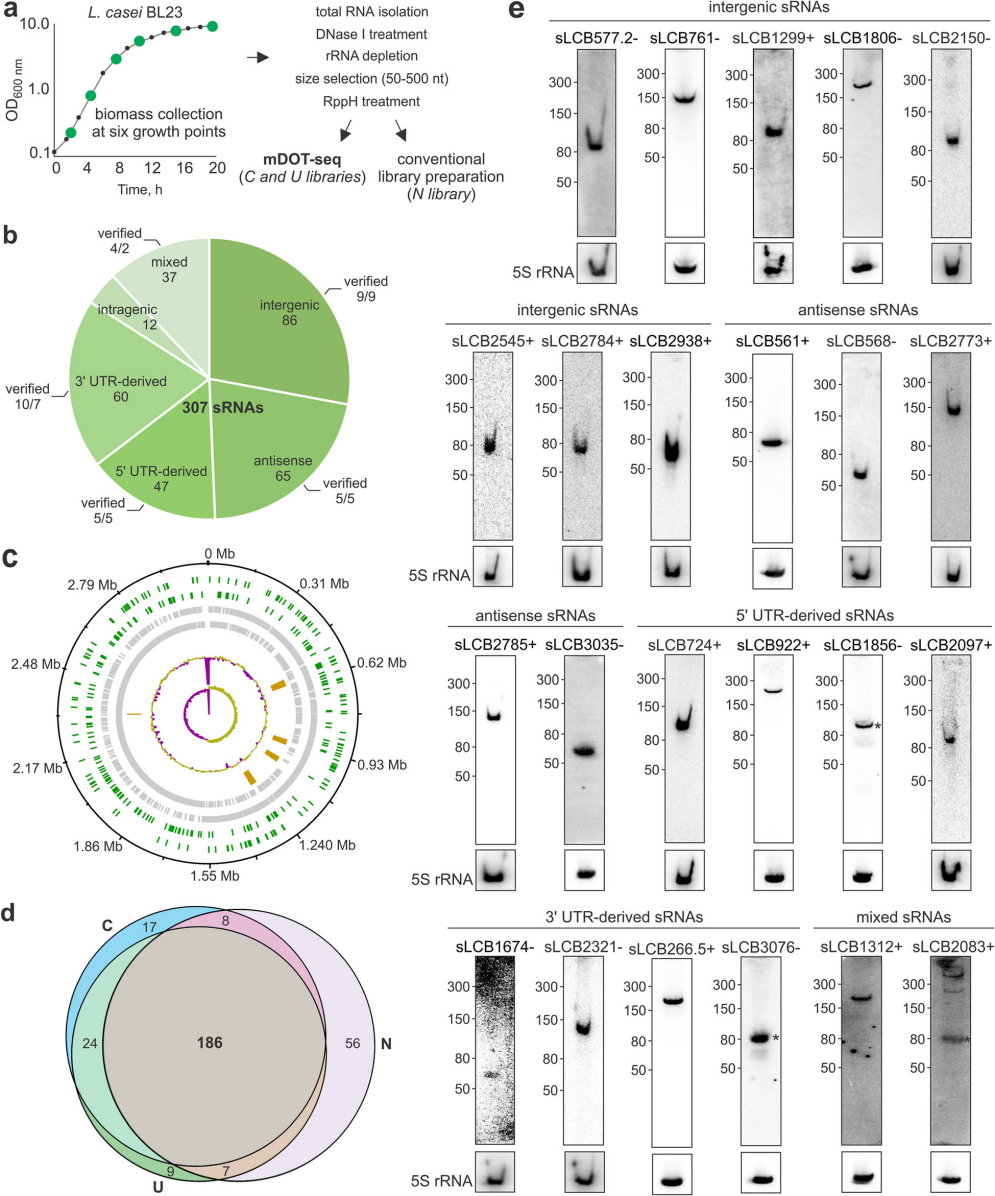
Profiling of small RNAs in *L. casei* BL23 using mDOT-seq. **a** A workflow for preparing the C, U and N sRNA libraries. **b** Group distribution of predicted sRNAs in *L. casei* BL23. sRNAs that could be assigned to more than one group were termed as “mixed” sRNAs. All validated sRNAs are listed in Table 1. **c** DNAplotter map of genomic positions of predicted sRNAs. Moving inwards from black circle denoting the genome of *L. casei* BL23, second and third green tracks represent the identified sRNAs on the forward and reverse strands respectively, fourth and fifth grey tracks — CDSs on forward and reverse strands accordingly, sixth orange track — prophage sequences and CRISPR region, seventh track denotes the GC content (%) and the inner most eighth track highlights the GC skew [(G − C)/(G + C)] of the genome with a window size of 10,000 bp and a step size of 200 bp (in both cases pink is for below the average and yellow green is for above the average). **d** Venn diagram of sRNAs identified in the C, U and N libraries. **e** Northern blot validation of sRNAs predicted in all three libraries. Asterisk marks sRNA where multiple bands are visible. 5S rRNA loading controls are provided at the bottom

To obtain comprehensive profiles of sRNAs expressed at different phases of the bacterial life cycle, we pooled *L. casei* BL23 cells harvested at six growth points (Fig. 4a). After depletion of rRNA, the extracted RNAs were size selected (50–500 nt) and treated with RNA 5′ Pyrophosphohydrolase (RppH) to preserve the entire small transcriptome of the cell, including primary transcripts with 5′ triphosphates and processed RNAs containing 5′ monophosphates. Following the developed mDOT-seq protocol, we prepared two strand-specific sequencing libraries C and U, for which 3′ alkyne-adapters containing ACTC and CUCC 5′ terminal sequences, respectively (Additional file 1: Fig. S9), were tethered to the pool of ssRNAs using click ligation. In parallel, a control library N was prepared by conventional 3′ adapter ligation following NEXTflex Small RNA-seq Kit v3 protocol. After Illumina sequencing of triplicate C, U and N libraries, we obtained an average of 15.7, 15.6 and 18.5 million high-quality paired-reads, respectively. Spearman correlation analysis of the mapped reads showed high correlations among biological replicates and the libraries, indicating a high reproducibility of the new method and its good general agreement with the conventional approach (Additional file 1: Fig. S10) [34]. A primary set of small non-coding RNAs in the transcriptome of *L. casei* was predicted by the APERO algorithm [35], which was manually processed to filter out transcripts with less than 15 average CPMs (counts per million) per library and those without distinguishable read coverage drop at their boundaries (with less than three times increase in read coverage within 2 nt at their 5′ and 3′ ends). In total, we identified 307 putative sRNAs expressed in *L. casei* BL23 that were classified into six groups based on their genomic context (Fig. 4b-d and Additional file 1: Fig. S11, Additional file 3: Table S2-7) as well as a group of transcripts encoded by Type II-A CRISPR-Cas system, in particular tracrRNA and some of processed crRNAs from CRISPR repeat-spacer array. The putative sRNAs included 86 intergenic, 107 untranslated region- (UTR-) derived, 65 antisense and 12 intragenic small RNAs, as well as 37 mixed sRNAs that could be assigned to more than one group, which is consistent with a number of non-coding RNAs generally observed in other bacteria [36]. Unlike protein-encoding genes (72% coding sequences, CDSs, are located on the leading strands in both directions from the replication origin of *L. casei* circular chromosome), intergenic sRNAs (56%) as well as sRNAs of all types (60%) exhibit weaker coding strand bias (Fig. 4c). Because genes encoded on the lagging strand are mutated more frequently [37], an increased mutagenesis rate is likely less deleterious for sRNAs and/or potentially beneficial for the adaptation to the environmental changes.

Out of 307 putative small non-coding RNAs in *L. casei* genome, 235, 226 and 257 were identified in C, U and N RNA sequencing data, respectively (Fig. 4d). A comprehensive comparison of short transcripts revealed that most 5′ end boundaries were reliably detected in all samples (Additional file 1: Fig. S12a). The libraries prepared by different techniques showed higher transcript heterogeneity at the 3′ ends (Additional file 1: Fig. S12b). Still, we consider that this bias is not inherent to a particular library preparation method because similar levels of 1-2 nt shorter transcripts were assigned in all libraries. 186 sRNAs were shared between all three libraries, and 201 overlapped when the N library prepared by the conventional method was compared to the libraries prepared by mDOT-seq (the sum of C and U), whereas 56 and 50 sRNAs were only predicted in the N and C + U sequencing libraries, respectively (Fig. 4d). We found that C + U exhibited only a few more putative intergenic (6 versus 4), antisense (16 vs 7), and 5′ UTR-derived sRNAs (12 vs 7) as compared to N. 3′ UTR-derived sRNA candidates were the most divergent: we detected 22 unique transcripts in N library prepared by the conventional method and only 7 in C + U libraries (Additional file 1: Fig. S13a). Moreover, all fifteen mixed group sRNAs found only in the N dataset could be attributed to 3′ UTR-derived sRNAs (6 in C + U). Thus, conventional RNA-seq tends to over-assign 3′ UTR-derived sRNAs. The logistic regression analysis of unique sRNAs revealed that candidates identified only in the N library are predisposed to form more stable secondary structures (of lower minimum free energy, *p* < 0.05) and contain shorter unstructured 3′ ends (*p* < 0.001) as compared to sRNAs identified only in the C + U libraries (Additional file 3: Table S8).

The cellular expression of selected sRNA candidates was experimentally verified by Northern blotting (Fig. 4e). The analysis of transcripts observed in all three libraries supports the sequencing data. All 23 tested sRNAs covering five different groups according to the genome context showed clearly detectable signals (Table 1 and Additional file 1: Fig. S14). In contrast, only 3 out of 8 candidate sRNAs predicted exclusively in the N library at the 3′ UTR were validated by Northern blotting (Table 1 and Additional file 1: Fig. S13b-d). This result implies that most of the unique 3′ UTR-derived sRNAs can be falsely predicted using conventional RNA sequencing. Thus, compared to the conventional library preparation protocol, the mDOT-seq method takes advantage of slightly better accuracy in detecting this group of transcripts. Overall, our experiments demonstrated the potential of the mDOT-seq technique as a powerful tool for small RNA analysis that is likely to become a viable alternative to the conventional RNA sequencing based on T4 RNA ligase 2 and hampered by its sequence and structure related biases [14–18].

**Table 1:** Candidate sRNAs analysed by Northern blotting (NB)

	Group	Name	Verified by NB
*sRNAs predicted in all libraries*	Intergenic	sLCB577.2−	+
		sLCB761−	+
		sLCB1299+	+
		sLCB1806−	+
		sLCB2150−	+
		sLCB2545+	+
		sLCB2784+	+
		sLCB2938+	+
	Antisense	sLCB561+	+
		sLCB568−	+
		sLCB2773+	+
		sLCB2785+	+
		sLCB3035−	+
	5′ UTR-derived	sLCB724+	+
		sLCB922+	+
		sLCB1856−	+
		sLCB2097+	+
	3′ UTR-derived	sLCB266.5+	+
		sLCB1674−	+
		sLCB2321−	+
		sLCB3076−	+
	Mixed - 3′ UTR-derived/5′ UTR-derived	sLCB1312+	+
	Mixed - 3′ UTR-derived/antisense	sLCB2083+	+
*sRNAs predicted only in the N library*	Intergenic	sLCB1735−	+
	5′ UTR-derived	sLCB616+	+
	3′ UTR-derived	sLCB3+	−
		sLCB518−	−
		sLCB1192−	−
		sLCB1398+	+
		sLCB1968−	+
		sLCB2960−	+
	Mixed - 3′ UTR-derived/antisense	sLCB1933−	−
		sLCB2710+	−

To understand the functions of predicted sRNAs, we searched the RNA families database (Rfam) [38, 39]. Although most candidates showed no similarity to the known RNA families suggesting their novelty, we could define the functions of 17 sRNAs. Three of them matched the housekeeping RNAs, including the catalytic RNA of RNase P (sLCB1642-), transfer-messenger RNR, tmRNA (sLCB1161-), and a signal recognition particle RNA (sLCB2400-). Other 14 predicted sRNAs belonged to nine RNA families of *cis*-regulatory elements (Additional file 3: Table S2). In addition, 2 sRNAs showed significant similarity to families of *Enterococcus* sRNAs (sLCB266.5+ and sLCB2938-). To evaluate the extent of taxonomical conservation of predicted sRNAs, we performed a blast search against six genomes from *Lactobacillus casei* group (LCG) of closely related bacteria [40] and nine genomes from more evolutionarily distant species of *Lactobacillaceae* family (Additional file 3: Table S9). As expected, the highest fraction of homologous sequences was identified in LCG, especially in the genomes of *L. paracasei* LcA (DN-11401, branded as *defensis*) and *L. paracasei* LcY (Shirota) isolated from Actimel and Yakult products marketed as probiotics, respectively [41]. All of the predicted sRNAs were present in *L. paracasei* LcA strain and showed sequence identity of more than 99% except for one sRNA (88.9%). Similar results were obtained in the search against *L. paracasei* LcY genome: only two sRNAs had sequence identity lower than 99%, and no similar sequences were identified for eight sRNAs (the incomplete assembly of *L. paracasei* LcY genome could account for the missing sRNAs). The obtained results confirm a close relatedness of these three strains indicating that the sRNA sequencing data generated in this study could potentially be translated to these broadly used probiotics. Homologues of sRNAs were also identified in *L. paracasei* ATCC (for 246 species), *L. paracasei subsp. paracasei* 8700:2 (242), *L. rhamnosus* GG (81) and *L. casei* DSM 20011 = JCM 1134 = ATCC 393 (76). In contrast, we could only detect sequences similar to 9 predicted sRNAs (including two housekeeping) in other tested strains of *Lactobacillaceae* family. Not a single homologue was identified in the genomes of *Lactobacillus acidophilus* NCFM and *Liquorilactobacillus nagelii* strain TMW 1.1827. Taken together, the obtained results indicate that the majority of sRNAs in *L. casei* BL23 are strictly group-specific.

## Discussion

### mDOT-seq as a new approach for RNA sequencing

In the current work, we show that a bioorthogonal chemo-enzymatic approach using small RNA methyltransferases can be successfully applied for covalent tethering of a suitable DNA adapter selectively to the 3′ end of target RNA permitting efficient RT reaction and strand-specific RNA-seq library preparation. Most importantly, with both DmHen1ΔC and AtHEN1, different RNA substrates are modified and later joined to the 3′ adapters in a sequence independent manner (Figs. 1b, c, 2b, c). This comes in contrast to the conventional RNA-seq library preparation when accurate identification of the 3′ terminal sequences is desirable. As several studies have shown, the inefficient primer ligation by T4 RNA ligase 2 stands out as the main reason of the often observed RNA sequence bias [14–18]. It has been reported that the conventional ligation reaction is prone to enriching or depleting certain RNAs from the sequencing library based on their secondary structure, number of unpaired nucleotides at the 3′ end or RNA-adapter co-fold [14, 18, 42]. The reported observations go in line with our results, showing that putative *L. casei* sRNAs detected only in the N library prepared using T4 RNA ligase 2 are more structured and have fewer free nucleotides at the 3′ end as compared to sRNAs identified only in the C and T libraries prepared by mDOT-seq (Additional file 3: Table S8). Interestingly, the detected false-positives in the N library are enriched in 3′ UTRs (Additional file 1: Fig. S13).

Installation of an orthogonal covalent linker bridging the target RNA with a sequencing adapter inevitably poses a question of how well it can be tolerated by available RT enzymes. We were surprised to learn that most of the RT variants were rather efficient in bypassing the examined DNA-RNA linker variants (Additional file 1: Fig. S3). Our systemic selection of the most efficient and accurate 3′ alkyne-adapter chemistries found that attachment of the linker chain at the second nucleotide supports efficient bypass synthesis of cDNA (Figs. 1d, 2d) permitting the lowest bias in miRNA quantification (Fig. 3c, Additional file 1: Fig. S8) [27]. A similar basic linker design has been successfully used for non-homologous tag-directed internal priming of the DNA polymerase action [21, 43], suggesting that similar underlying principles may govern the strand extension reactions on both RNA (A-helix) and DNA (B-helix) templates by distinct polymerase/transcriptase enzymes. This proposed structural feature of the priming reaction is a proper positioning of the 3′ terminal nucleotide of the template strand aided by the following major factors: the flexible chemical linker, stacking interactions with the 5′ terminal nucleotide of the tethered adapter [21] and, possibly, interaction with certain residues in the catalytic site of the RT. In addition, we found that the identity of the first nucleotide at the 5′ end of the adapter appears to play some role as nucleobases possessing an exocyclic amino group in the major groove (C and A) resulted in the highest number of reads identified (Fig. 3d). The structural basis of this phenomenon is not clear.

As the results obtained with the selected 2C and 2U adapters are highly similar with respect to both the reproducibility (Additional file 1: Fig. S10) [34] and the breadth of sRNA capture (Fig. 4d), we believe that either adapter or their combination can well be used for analytical applications. On the other hand, since a suboptimal, M-MuLV, reverse transcriptase was used in the miRXplore Universal Reference sequencing experiment, the observed sRNA capture bias (Additional file 1: Fig. S6, S7) possibly stems from this particular step in the RNA library preparation. It is therefore likely that this minor bias can be reduced or even eliminated by selection of better-performing reverse transcription enzyme and further refinement of the procedure.

### mDOT-seq profiling of sRNAs in probiotic *Lactobacillus casei*

Starting from the miRXplore Universal Reference and expanding to the sRNA transcriptome of *L. casei* BL23 we prove that mDOT-seq can be used for the identification and quantitation of RNAs ranging from 16 nt to at least 500 nt in length. To the best of our knowledge, this is the first thorough characterisation of a *L. casei* BL23 sRNA transcriptome, as up to date only one report has described sRNAs in the *Lactobacillus* genus [44]. In total, we identify 307 putative sRNAs with more than a third deriving from 5′-UTR, 3′-UTR and intragenic loci (Fig. 4b). This finding goes in line with the increasing recognition of the prevalence of small transcripts from the aforementioned regions [36, 45, 46].

As more than a fifth of the predicted sRNAs are conserved among the analysed representative strains of *Lactobacillus casei* group (LCG) (Additional file 3: Table S9), we envision that the results obtained in this study will serve as a valuable resource for achieving a better understanding of LCG physiology or even their improved applications in industry and medicine. LCG are among the most studied probiotics with high potential in prophylactics and therapeutics. Administration of LCG improves the balance of gut microbiota and has a positive effect on the brain function, weight management in obese patients and control of infectious and autoimmune diseases, including cancer. As probiotics LCG encounters numerous stressors both before administration and in gastrointestinal tract and respond to them by subtle alterations in their metabolism [40, 47]. The importance of sRNAs in stress response pathways are well appreciated in broadly characterised enterobacteria [48]. The produced data will serve as a useful source and prime the elucidation of the roles of sRNAs in stress response in LCG bacteria.

RNA methyltransferases are increasingly applied for analysis of both transcriptome and epitranscriptome [4, 5, 11, 49]. We envision that mDOT-seq can be instrumental for detecting transcriptomic profiles of total ssRNAs (with DmHen1) and 21-24 dsRNAs (with AtHEN1) in situ or for analysing the methylome of piRNAs (using DmHen1) and miRNA/siRNA duplexes (with AtHEN1) in vivo. In this context, mDOT-seq would bring the advantage of direct identification of modifiable nucleotide in live cells. From a broader perspective, we suggest that the general methyltransferase-tagged RNA sequencing approach, successfully exemplified here using Hen1 methyltransferases, could adapt other RNA methyltransferases for transcriptome wide identification and profiling of their modification sites at a single-base resolution.

## Conclusions

Progress in innovative bioorthogonal ligation methodologies provides a plethora of opportunities for advanced biomolecular analysis in vitro, in live cells and in whole organisms. Here, we exploited two *S*-adenosyl-L-methionine dependent RNA methyltransferases for the development of a new chemo-enzymatic approach — methyltransferase-Directed Orthogonal Tagging and RNA sequencing, mDOT-seq — as an attractive alternative to conventional T4 RNA ligase-based RNA-seq library preparation which suffers from RNA sequence and structure related biases. The dsRNA specific AtHEN1 and ssRNA modifying DmHen1 were applied for sequence independent transfer of a six-carbon linear chain carrying a terminal azide group from a synthetic AdoMet analogue onto the 3′ terminal RNA nucleotide for subsequently adapter-tagging using copper-catalysed azide-alkyne cycloaddition. We demonstrate that the resulting RNA-DNA linkage with a central triazole ring can be efficiently traversed by certain reverse transcriptases during the synthesis of cDNA. To our knowledge, this is the first application of a bioorthogonal click reaction mediated 3′ adapter ligation for RNA-seq library preparation. mDOT-seq was successfully applied for the identification of 16–28 nt miRNAs and 50–500 nt small non-coding bacterial sRNAs thus resulting in the characterisation of the sRNA profile of the probiotic *Lactobacillus casei* BL23. We envision that the presented mDOT-seq technique could be easily adapted to a wide variety of other RNA methyltransferases or could be expanded to facilitate the analysis of (epi)transcriptome not only in vitro but also in living cells.

## Methods

### ssRNA modification using DmHen1ΔC, click reaction and reverse transcription

DmHen1∆C was expressed and purified as described in Mickutė et al. [10]. All alkylated DNA oligonucleotides were purchased from Base Click while RT primer and N21 RNA was ordered from Metabion. Where needed nucleic acids were labelled at the 5′ end using ATP, [γ-^32^P] (PerkinElmer) and T4 PNK (Thermo Fisher Scientific) following the manufacturer’s recommendations. For RNA modification with azide group, 0.2-10 μM of N21 RNA (random sequence 21-mer) was mixed with 0.1–0.2 mM Ado-6-azide and 2 μM of DmHen1∆C in the ssRNA modification reaction buffer containing 10 mM Co^2+^ in the form of CoCl_2_ salt, 10 mM Tris-HCl (pH 7.4), 50 mM NaCl, 5% glycerol, 0.2 mM DTT, 0.1 mg/ml BSA and 0.04 U/μl RiboLock RNase inhibitor (Thermo Fisher Scientific) and incubated for 30–60 min at 37 °C. Reactions were quenched with Proteinase K as described in Mickutė et al. [10], and RNA was precipitated after the addition of 1/10 volume of 3 M NaOAc, pH 5.2:20 mg/ml glycogen (19:1) and 2.5 volumes of 96% ethanol and dissolved in water. Click reactions were performed in 55% DMSO containing 0.025-0.2 μM RNA-azide, 1.25-10 μM 3′ alkyne-adapter/RT primer (5′-GCCTTGGCACCCGAGAATTCCA-3′) (annealed at equimolar amount in Annealing buffer of 7.5 mM HEPES-KOH, 0.5 mM MgCl_2_, 25 mM KCl, pH 7.4) and 3.3 mM of freshly prepared CuBr-TBTA for 30 min at 45 °C. As for reverse transcription, the concentrations of reagents in click reaction were modified so that 20 μM of RNA-azide was mixed with 5 μM of 3′ alkyne-adapter/RT primer and incubated for 45 min. Following precipitation, 10 nM of RNA-DNA conjugate was reverse transcribed in provided reaction buffer containing 1U/μl RiboLock RNase inhibitor, 0.25 mM of dNTP and 10 U/μl of RevertAid, RevertAid H Minus, Maxima or Maxima H Minus Reverse Transcriptase (Thermo Fisher Scientific) at 38 °C or M-MuLV Reverse Transcriptase (New England Biolabs) at 42 °C for 2–4 h. The reaction was terminated by heating at 70 °C for 10 min. All samples were analysed on 13% denaturing polyacrylamide gel (dPAG) as described in Mickutė et al. [10].

### dsRNA modification using AtHEN1, click reaction and reverse transcription

AtHEN1 was expressed and purified as described in Osipenko et al. [13]. All alkylated DNA oligonucleotides were purchased from Base Click, while RT primer, miRNAs and non-alkylated DNAs were ordered from Metabion (Additional file 4: Table S10). Where needed nucleic acids were labelled at the 5′ end using ATP, [γ-^32^P], and annealed to a complimentary strand by incubating at 85 °C for 3 min with subsequent cool down by − 0.6 °C/min to 4 °C resulting in RNA/RNA or RNA/DNA duplexes with 2 nt 3′ overhangs. For RNA modification with azide group 0.2-10 μM of miRNA/miRNA* or miRNA/DNA substrate(-s) was mixed with 0.3–400 μM Ado-6-azide and 0.25-2 μM of AtHEN1 in the dsRNA modification reaction buffer (10 mM Tris-HCl, pH 7.5, 50 mM NaCl, 0.1 mg/ml BSA and 0.04 U/μl RiboLock RNase inhibitor (Thermo Fisher Scientific)) and incubated for 1–2 h at 37 °C. Reactions were quenched with Proteinase K and RNA was precipitated as described above. Click reactions, reverse transcription and analysis on dPAG were performed as described earlier.

### miRXplore Universal Reference RNA library preparation

2.5 μM of miRXplore Universal Reference (Miltenyi Biotec) RNA was heated to 82.5 °C in the presence of 0.1 mM EDTA for 2 min and immediately placed on ice for 5 min. Then, RNA sample was split in two and libraries were prepared in duplicate for each 3′ alkyne-adapter. 2 μM of DmHen1∆C was used to modify 0.2 μM of miRXplore RNA in the ssRNA modification reaction buffer as described earlier. Proteinase K treatment and RNA precipitation was followed by click reaction during which 0.4 μM of RNA-azide was incubated with 10 μM of 3′ alkyne-adapter/RT primer and precipitated afterwards. 0.35 pmol of RNA-DNA conjugate was used for RNA library preparation according to the NEXTflex Small RNA-seq Kit v3 (PerkinElmer) starting from Step B with the following modifications: at Step D, ¼ dilution of NEXTflex 5′ 4 N adapter was used; at Step G, 18 cycles of PCR were performed; and Step H1 was proceeded with Step H2. The size distribution of the library was evaluated by capillary electrophoresis on the Agilent 2100 Bioanalyzer using Agilent High Sensitivity DNA Kit (Agilent Technologies) and quantified via a qPCR reaction using KAPA Library Quantification Kit (Roche) on a Rotor-Gene Q instrument. All RNA libraries were pooled and sequenced at Lexogen facilities using NextSeq 500/550 High Output Kit to obtain 1 × 75 nt single-end reads.

### miRXplore Universal Reference sequencing data analysis

The quality of raw sequencing reads was evaluated using FASTQC program (https://www.bioinformatics.babraham.ac.uk/projects/fastqc/). Reads that were too short (less than 10 bases), had no adapters or contained Ns in the sequence were removed from further analysis using cutadapt [50]. Remaining reads were deduplicated using custom Unix script and size filtered to fit a range of 15 to 34 nt. To count the reads corresponding to each miRNA, we created custom reference libraries from 1005 distinct miRNAs by extracting only those that differed by their n-x nucleotides from the 5′ end (where n is equal to the length of the miRNA and x denotes the number of nucleotides cropped from its 3′ end, 0 ≤ n ≤ 5). These reference libraries and a custom Unix script was used to calculate the amount of individual miRNA in each sample by exact match. All statistical calculations were performed using R 3.5 [51].

Data analysis pipeline is provided in the following scheme.

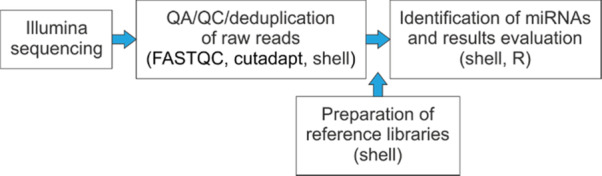



### *L. casei* sRNR library preparation

*Lactobacillus casei* BL23 (a kind gift of Marie-Pierre Chapot-Chartier and Saulius Kulakauskas from Micalis Institute, INRA, France) was grown in 200 ml of BD™ Difco™ Lactobacilli MRS Broth at 37 °C until it reached six different growth points in early, mid and late exponential and stationary growth phases where equal amount of bacteria were collected from three biological replicates, mixed with 1/8 volume of ice-cold STOP solution (95% ethanol and 5% acid phenol), centrifuged and stored at − 80 °C. Frozen cells from all six growth stages were mixed and ground to a fine powder in liquid nitrogen. About 100 mg of ground powder was used for the total RNA extraction with RNAzol RT (RN 190) (Molecular Research Center, Inc.) according to the manufacturer’s recommendations. The quality of extracted RNA was evaluated using Agilent RNA 6000 Nano Kit (Agilent Technologies). For sRNA library preparation total RNA was treated with DNase I, RNase free (Thermo Scientific) following the manufacturer’s recommendations and recovered from the reaction using RNA Clean & Concentrator-25 Kit (Zymo Research). rRNAs were depleted using the modified RiboMinus Transcriptome Isolation Kit, bacteria (Invitrogen) where 2 μl of RiboMinus Probes and 2 μl of 100 μM mix of three probes specific to 5S rRNA were used (Additional file 4: Table S11). After ethanol precipitation RNA was subjected to size selection as 50–500 nt RNA was cut out of 8% denaturing PAA gel. Gel slices were crushed with a disposable pestle and RNA was eluted in 500 μl of Elution buffer (0.5 M NH_4_OAc, 0.1% SDS, 0.1 mM EDTA) in 4 h in a 25 °C incubator rotating at 300 rpm. The eluate was collected using Corning Costar Spin-X Centrifuge Tube Filters (Merck) and RNA precipitated with 1/10 volume of 3 M NaOAc, pH 5.2:20 mg/ml glycogen (19:1) and 2.5 volumes of 96% ethanol. After treatment with RNA 5′ Pyrophosphohydrolase (RppH) (New England Biolabs) according to the manufacturer’s recommendations and further recovery with RNA Clean & Concentrator-5 Kit (Zymo Research) for mDOT-seq 1.6 μM of RNA was heated at 82.5 °C for 2 min in the presence of 0.1 mM of EDTA and immediately transferred on ice for 5 min. As with miRXplore Universal Reference library preparation, 0.2–0.3 μM of RNA were modified with azide group and 0.2 μM of azide-RNA was used for click reaction. Two picomoles of RNA-DNA conjugate was further processed according to the NEXTflex Small RNA-Seq Kit v3 (PerkinElmer) starting from the Step B with couple modifications listed: at Step D, ¼ dilution of NEXTflex 5′ 4 N adapter was used; at Step E, incubation at 42 °C was extended to 60 min; at Step G, 15 PCR cycles were performed; and Steps F and H1 were carried out as described in alternative protocol for Preparing Libraries without Size Selection. For N libraries, 2 pmol of RppH treated RNA was processed according to the NEXTflex Small RNA-Seq Kit v3 (PerkinElmer) with modifications in Steps E, F and H1 identical to mDOT-seq protocol except for Step G were only 12 PCR cycles were performed. The quality and concentration of libraries was evaluated as indicated above. All libraries were pooled and sequenced at Lexogen facilities using NextSeq 500/550 Mid Output Kit to obtain 2 × 75 paired-end reads.

### *L. casei* sRNA sequencing data analysis

The quality of raw sequencing reads was evaluated using FASTQC program v0.11.9 (https://www.bioinformatics.babraham.ac.uk/projects/fastqc/). Low-quality reads or those having Ns were filtered out using cutadapt v2.9 [50], and data was deduplicated using BBTools package v38.41 (https://sourceforge.net/projects/bbmap/). Deduplicated dataset was quality (PHRED threshold 20) and length (minimum length 30) filtered using Trim Galore! v0.6.5 (https://www.bioinformatics.babraham.ac.uk/projects/trim_galore/). Adaptor sequences were removed using UMI tools v1.0.1 [52]. Resulting reads were mapped using hisat2 v2.2.0 [53] on a *L. casei* reference genome (NC_010999.1). Mapped data were subjected to APERO v1.0.3 [35] analysis (using standard parameters except for the wmax = 3 and min_read_number = 10) and followed by a manual crosscheck. Transcripts of > 3x increase in read coverage within 2 nt at their 5′ and 3′ ends in at least one of three replicates were considered as potential sRNAs. In cases where based on the required increase in read coverage slightly different coordinates were assigned to the same sRNA in separate libraries, for the common list of sRNAs, the widest ones were picked. Abundance of predicted sRNAs was evaluated using featureCounts from RSubread v1.32.4 package [54] and normalised using TMM method (edgeR v3.24.3 package) [55]. Only sRNAs with an average CPM of ≥ 15 in at least one library were included in further analysis. Promoters and terminators of candidate sRNAs were predicted using BPROM and FindTerm [56] using standard search parameters. sRNAs homologue sequences in other genomes were identified and aligned using standalone BLAST 2.9.0+ [57] and needle from EMBOSS package v6.6.0.0 [58] using standard search and alignment parameters. RFAM families were identified using Infernal cmscan at EBI (https://www.ebi.ac.uk/Tools/rna/infernal_cmscan/) [59]. sRNAs’ structures and minimal free energies were calculated using RNAfold program from Vienna RNA package v2.4.16 [60]. Visual genomic representations of potential sRNAs were created using Integrative Genomic Viewer [61]. Custom Unix script was used to prepare the data and artificially fill in insert and concatenate paired end reads. All statistical calculations were performed using R 3.5 [51], except for logistic regression analysis which was done using past3 software [62].

Pipeline for sequencing data analysis and identification of putative sRNAs is provided in the following scheme.

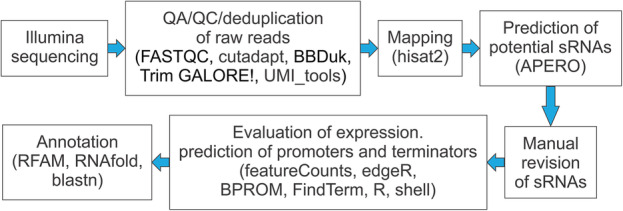



### Northern blot analysis

To validate predicted sRNAs, 10 μg of total RNA was fractionated on an 8% polyacrylamide/7 M urea gel and transferred to SensiBlot™ Plus Nylon Membrane (Thermo Scientific) by electroblotting at 5 V for 2 h using *V20*-*SDB* semi-dry blotter (Fisherbrand). Blots were UV-cross-linked by irradiation at 254 nm, 25 J in a crosslinker (UVITEC Cambridge) and prehybridised for 2 h with a Hybridization buffer (0.5 M Na_2_HPO_4_ × 2H_2_O, 7% SDS, 1 mM EDTA, 1% (w/v) BSA, pH 7.2). Membranes were incubated overnight at 42 °C with γ^32^P-ATP end-labelled oligodeoxyribonucleotides specific to certain sRNAs (Additional file 4: Table S12). Hybridised blots were washed three times in a Wash buffer 1 (2 × SSC, 0.1% SDS), 2 (1 × SSC, 0.1% SDS) and 3 (0.1 × SSC/0.1% SDS) for 15 min at 42 °C and exposed to phosphor imaging plates (Fujifilm). Signals were visualised with a FLA-5100 Image Reader (Fujifilm). The same membranes were used to detect 5S rRNA after stripping of radiolabelled probe with boiling 0.1% SDS twice. Stripped blots were washed 3 times with water and hybridised with 5S rRNA probe as described above.

## Supplementary Information

**Additional file 1: Fig. S1.** The chemical structures of alkyne-modified nucleotides and nucleosides. **Fig. S2.** Alkyne-adapter is efficiently attached to RNA-azide of nanomolar concentration. **Fig. S3.** cDNA synthesis through a conjugation linker using different reverse transcriptases. **Fig. S4.** Different miRNAs are efficiently modified using Ado-6-azide cofactor and AtHEN1. **Fig. S5.** Comparison of libraries prepared using different 3′ alkyne-adapters. **Fig. S6.** Effects of various factors on miRNA representation in mDOT-seq libraries. **Fig. S7.** Deviation of observed nucleotides frequencies from expected values in 3′-terminal section (16 positions) of identified miRNAs. **Fig. S8.** Evaluation of the miRNA quantification accuracy using different 3′ alkyne-adapters. **Fig. S9.** Sequence of 3′ alkyne-adapters applied for *Lactobacillus casei* sRNAs sequencing. **Fig. S10.** Spearman correlation among biological replicates of the *L. casei* libraries. **Fig. S11.** Classification of sRNAs into groups based on their genomic context. **Fig. S12.** The amount of predicted sRNAs with shorter 5′ or 3′ ends is similar among different libraries. **Fig. S13.** Putative 3′ UTR-derived sRNAs tend to be incorrectly identified in the N library. **Fig. S14.** Read coverage plots of experimentally verified sRNAs identified in all three libraries. **Table S1.** The number of miRNA species captured in sequenced libraries.

**Additional file 2.** The data underlying published graphs.

**Additional file 3: Table S2.** List of predicted sRNAs and their main features. **Table S3.** List of predicted sRNAs originating from more than one loci and their main features. **Table S4.** Promoters of predicted sRNAs. **Table S5.** Terminators of predicted sRNAs. **Table S6.** Detailed genomic context of predicted sRNAs. **Table S7.** Detailed genomic context of predicted sRNAs originating from more than one loci. **Table S8.** Structural characteristics of predicted sRNAs. **Table S9.** Conservation of predicted sRNAs in *Lactobacillaceae* family.

**Additional file 4: Table S10.** The list of RNA and DNA oligonucleotides used for labelling of double-stranded substrates with AtHEN1. **Table S11.** The list of *L. casei* BL23 5S rRNA specific probes used for rRNA depletion. **Table S12.** DNA oligonucleotides used for Northern blot analysis.

## Data Availability

All data generated or analysed during this study are included in the published article, its supplementary information files and publicly available repositories. RNA-seq data generated for this study are deposited on GEO under accession GSE166932.
