# Supplementary material for: Methyltransferase-directed orthogonal tagging and sequencing of miRNAs and bacterial small RNAs
Source: BMC Biol. 2021 Jun 22;19:129. doi: 10.1186/s12915-021-01053-w (PMC8220740; doi:10.1186/s12915-021-01053-w)
Supplement: Supplementary file 1 — Additional file 1: Fig. S1. The chemical structures of alkyne-modified nucleotides and nucleosides. Fig. S2. Alkyne-adapter is efficiently attached to RNA-azide of nanomolar concentration. Fig. S3. cDNA synthesis through a conjugation linker using different reverse transcriptases. Fig. S4. Different miRNAs are efficiently modified using Ado-6-azide cofactor and AtHEN1. Fig. S5. Comparison of libraries prepared using different 3′ alkyne-adapters. Fig. S6. Effects of various factors on miRNA representation in mDOT-seq libraries. Fig. S7. Deviation of observed nucleotides frequencies from expected values in 3′-terminal section (16 positions) of identified miRNAs. Fig. S8. Evaluation of the miRNA quantification accuracy using different 3′ alkyne-adapters. Fig. S9. Sequence of 3′ alkyne-adapters applied for Lactobacillus casei sRNAs sequencing. Fig. S10. Spearman correlation among biological replicates of the L. casei libraries. Fig. S11. Classification of sRNAs into groups based on their genomic context. Fig. S12. The amount of predicted sRNAs with shorter 5′ or 3′ ends is similar among different libraries. Fig. S13. Putative 3′ UTR-derived sRNAs tend to be incorrectly identified in the N library. Fig. S14. Read coverage plots of experimentally verified sRNAs identified in all three libraries. Table S1. The number of miRNA species captured in sequenced libraries. [file 12915_2021_1053_MOESM1_ESM.pdf]

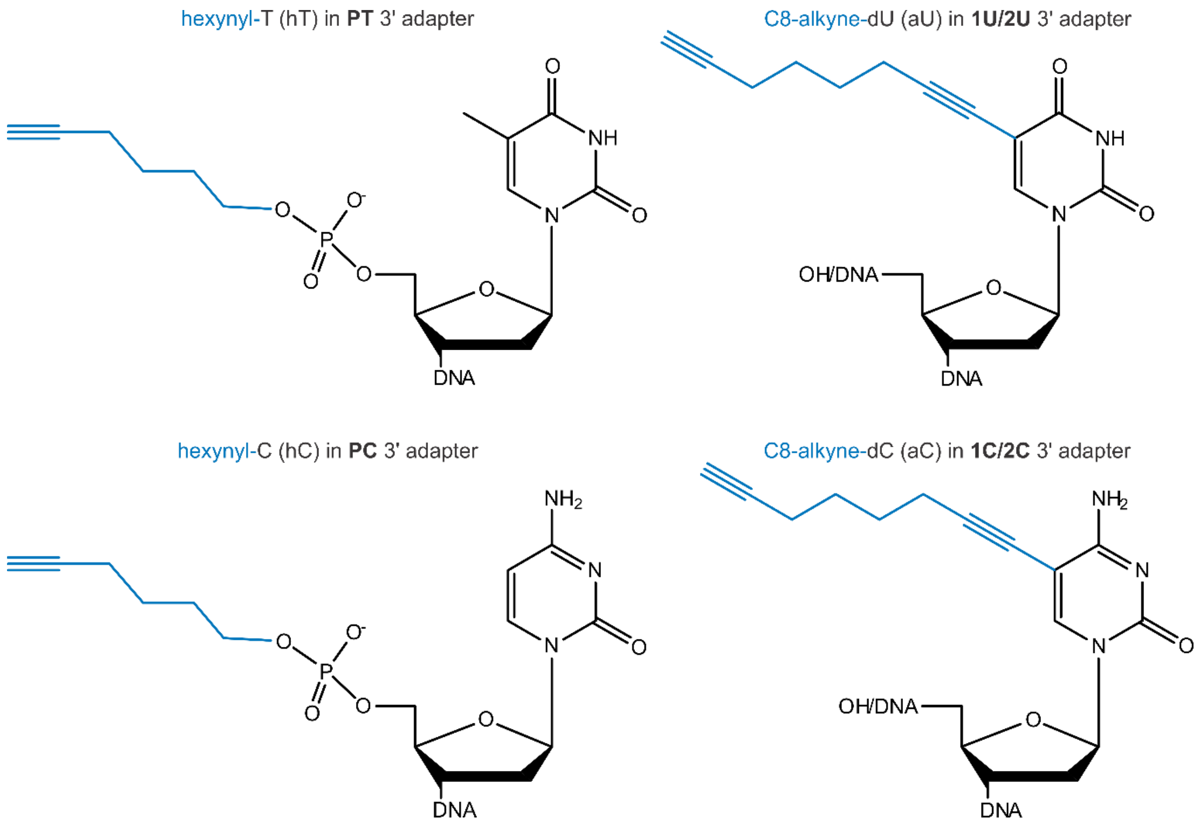

Fig. S1 The chemical structures of alkyne-modified nucleotides and nucleosides incorporated into 3' adapters by chemical synthesis. Aliphatic side chains with functional groups are coloured in blue.

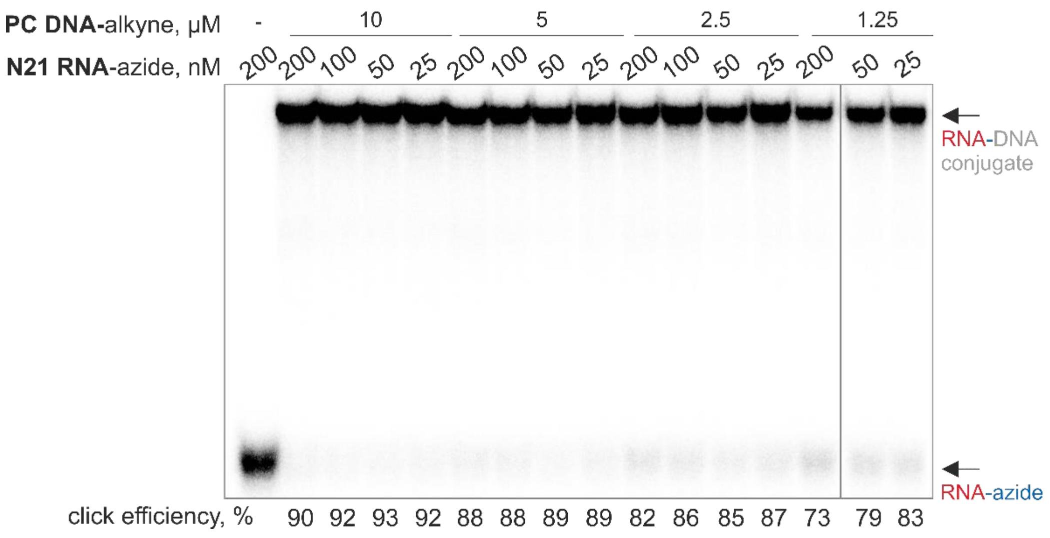

Fig. S2 Alkyne-adapter is efficiently attached to RNA-azide of nanomolar concentration. Reactions were performed using indicated concentrations of 3' alkyne-adapter and DmHEN1ΔC pre-modified N21 RNA-azide in the presence of 3.3 mM Cu(I)Br-TBTA and 55% DMSO.

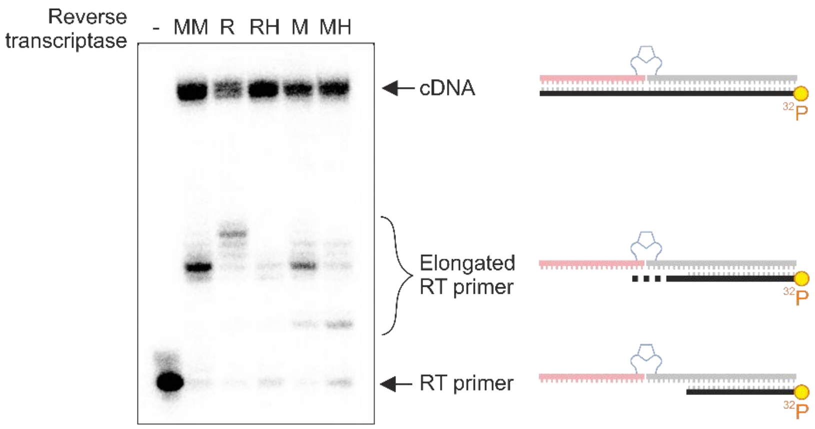

Fig. S3 cDNA synthesis through a conjugation linker using different reverse transcriptases. 10 µM of N21 RNA was incubated with 2 µM DmHen1 and 200 µM Ado-6-azide for 1 hour at 37 ºC. Samples were treated with proteinase K, precipitated in ethanol and incubated with 2U alkylated DNA/^{32}P-RT primer for click reaction and reverse transcribed under subsequent conditions: 10 nM RNA-DNA conjugate, 0.25 mM dNTPs and 10 u/µl of respective reverse transcriptase for 4 hours at 38 ºC with the exception of 42 ºC for M-MuLV. MM – M-MuLV RT, R – RevertAid RT, RH – RevertAid H Minus RT, M – Maxima RT, MH – Maxima H Minus RT.

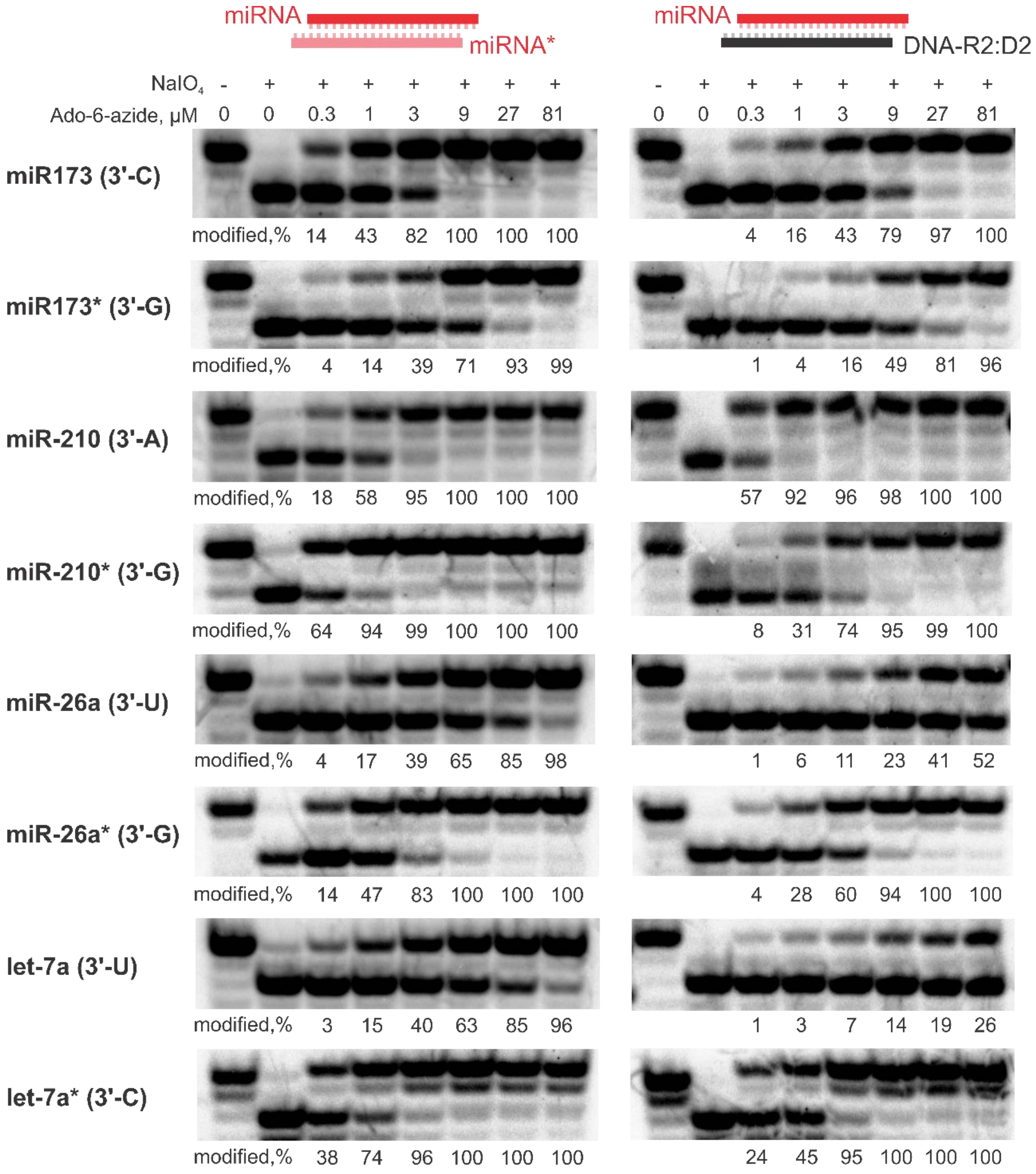

Fig. S4 Different miRNAs are efficiently modified using Ado-6-azide cofactor and AtHEN1. 0.1 µM of ^{33}P-labeled miR173, miR173*, miR-210, miR-210*, miR-26a, miR-26a*, let-7a or let-7a* were annealed with 0.12 µM of complementary miRNA (left column) or DNA (right column) and incubated with 0.25 µM AtHEN1 and 0.3-81 µM Ado-6-azide for 1 hour at 37 ºC. Samples were treated with sodium periodate ($NaIO_4$), separated on dPAG and the percentage of modified RNA was calculated using MutiGauge v.3.0 software. Lower bands depict non-modified RNA, higher bands – modified RNA or $NaIO_4$ non-treated control.

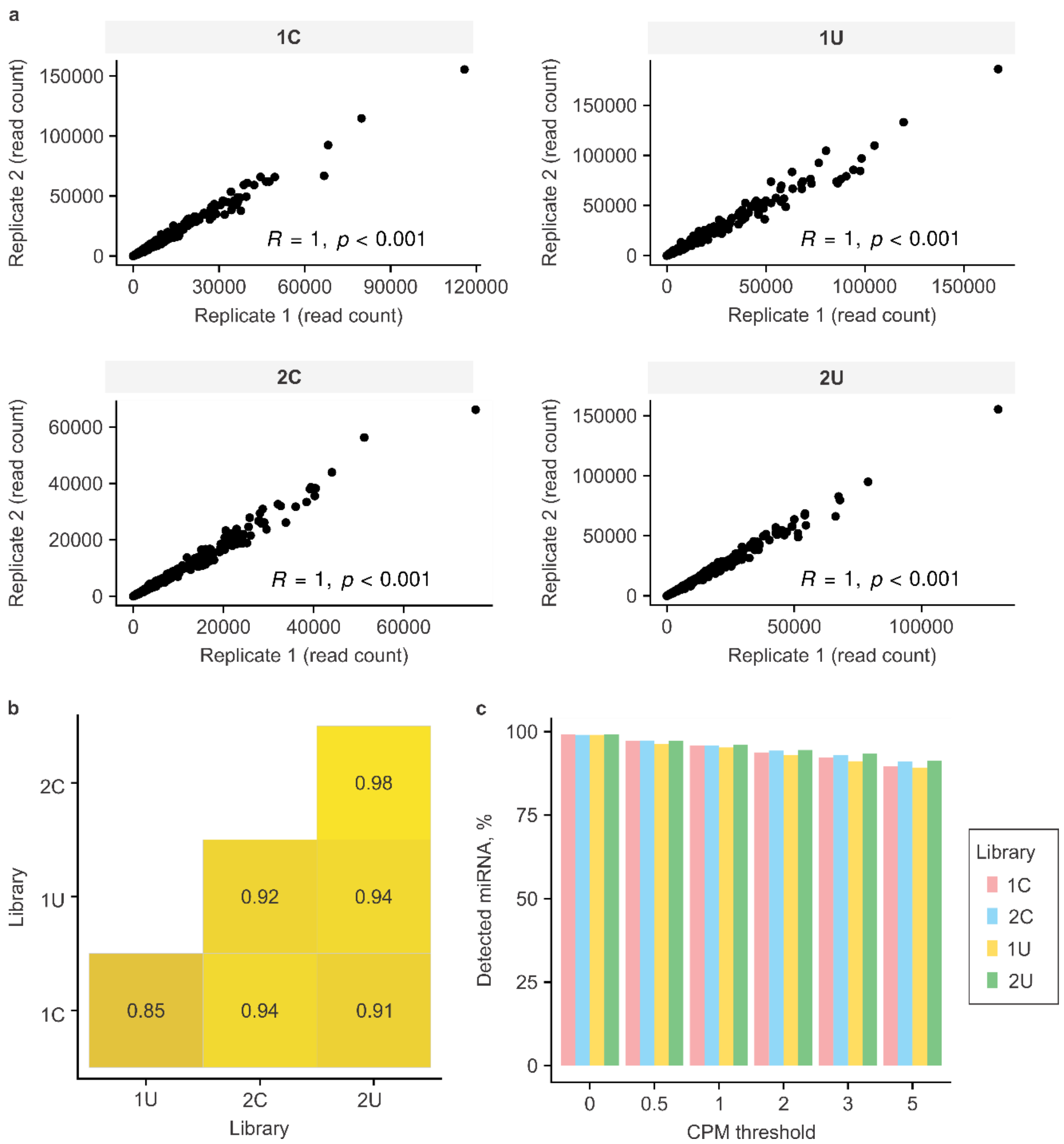

Fig. S5 Comparison of libraries prepared using different 3' alkyne-adapters. **a** Correlation between experimental replicates. Counts of the raw reads representing individual unique full-length miRNAs were used to calculate the Spearman correlation between the libraries. **b** The correlation between libraries prepared using different 3' alkyne-adapters. Read counts of full-length miRNAs from the experimental replicates were pooled and used to calculate Spearman correlation between distinct libraries. **c** Increasing the CPM threshold results in the highest fraction of detected miRNAs in 2U and 2C libraries. Reference sequences cropped of the last two nucleotides at the 3' end were used to quantify the miRNA reads. The data underlying the presented graphs are in Additional file 2.

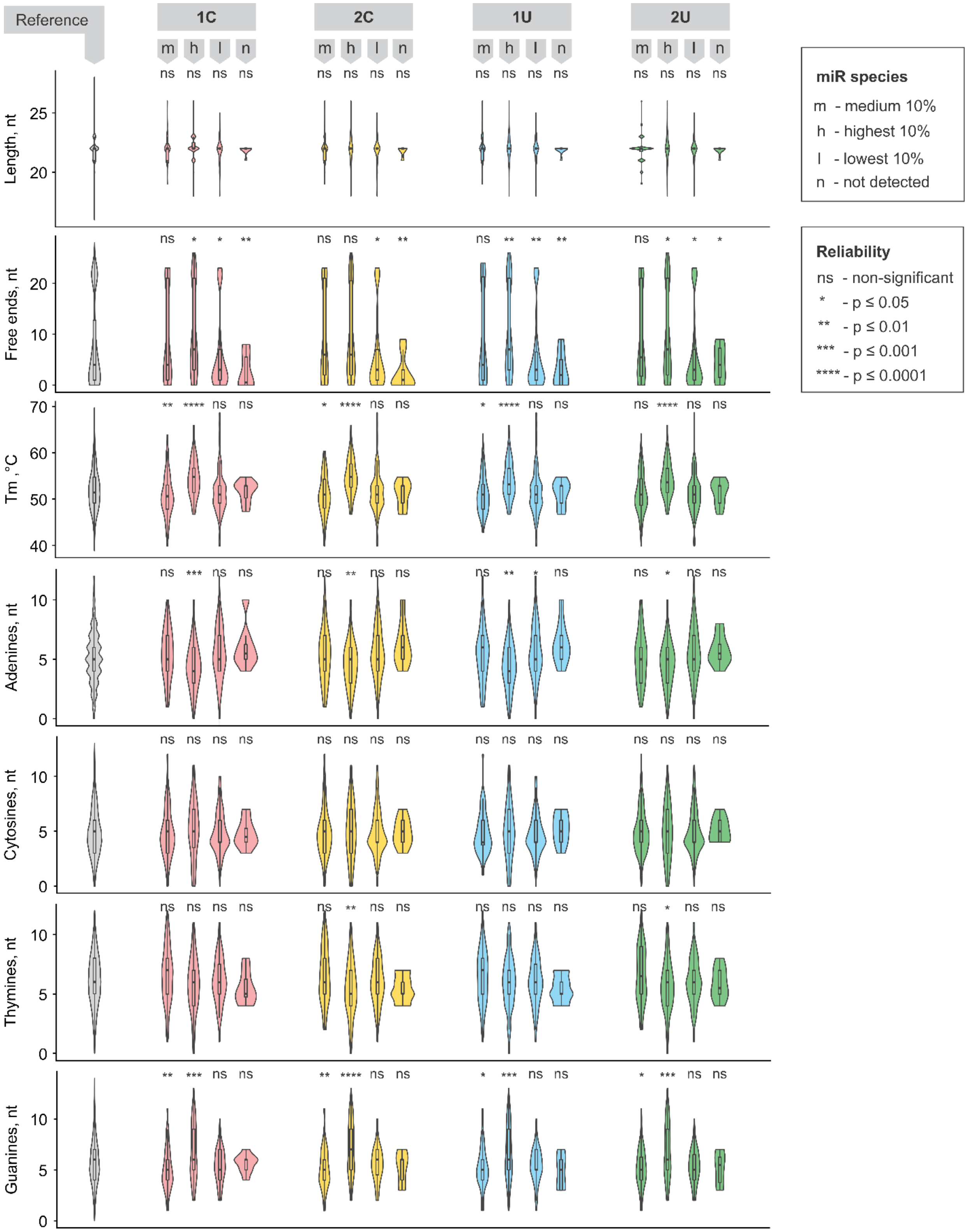

Fig. S6 Effects of various factors on miRNA representation in mDOT-seq libraries. Four subsets of miRNAs were selected (10% of highly-, medium-, low-represented and not detected) and miRNA length, Tm, number of free nucleotides at 3' end and G/A/T/C amount per each group was calculated. T-test was performed to evaluate differences between each group and a reference group. Asterisks indicate statistical significance as described in the legend. Reference sequences cropped of the last two nucleotides at the 3' end were used to identify the miRNA reads. The data underlying the presented graphs are in Additional file 2.

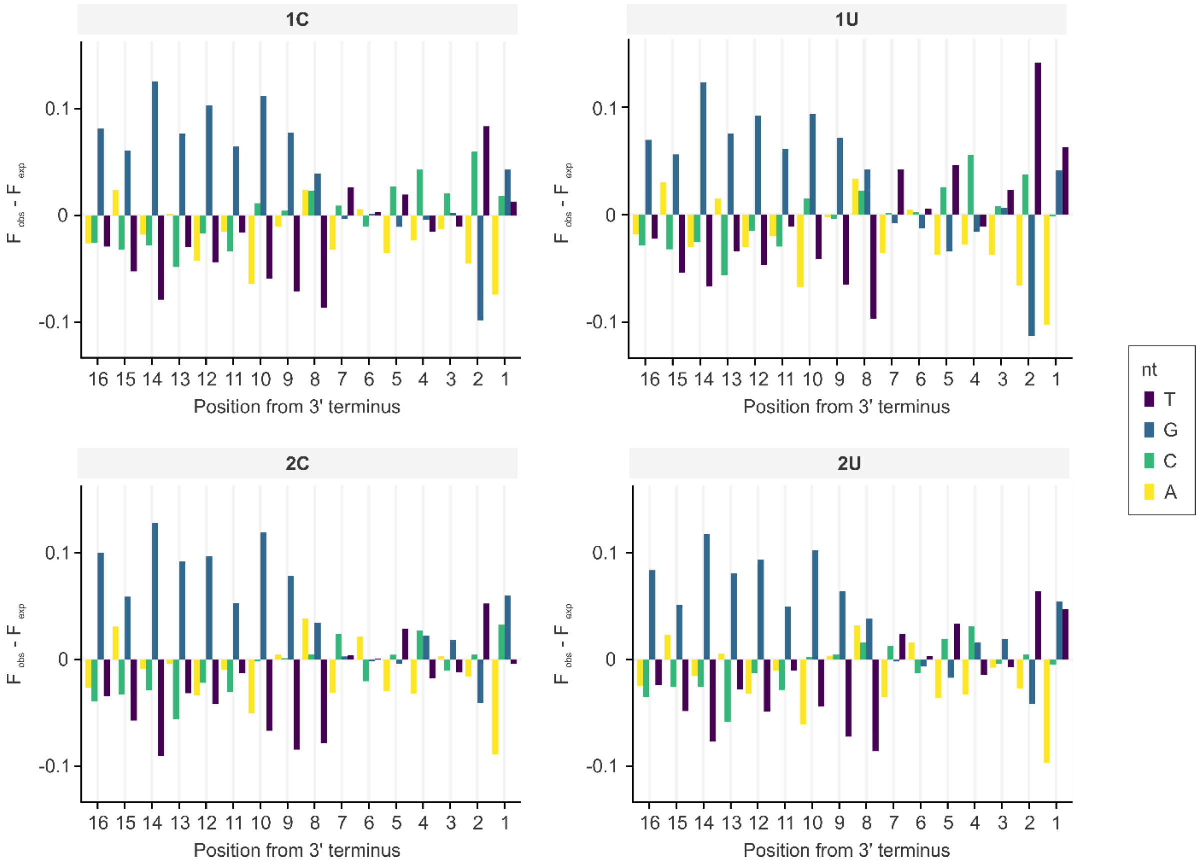

Fig. S7 Deviation of observed nucleotides frequencies from expected values in 3'-terminal section (16 positions) of identified miRNAs. The fraction of each nucleotide in the miRXplore RNA pool at a particular position was subtracted from the observed one. Reference sequences cropped of the last two nucleotides at the 3' end were used to identify the miRNA reads. The data underlying the presented graphs are in Additional file 2.

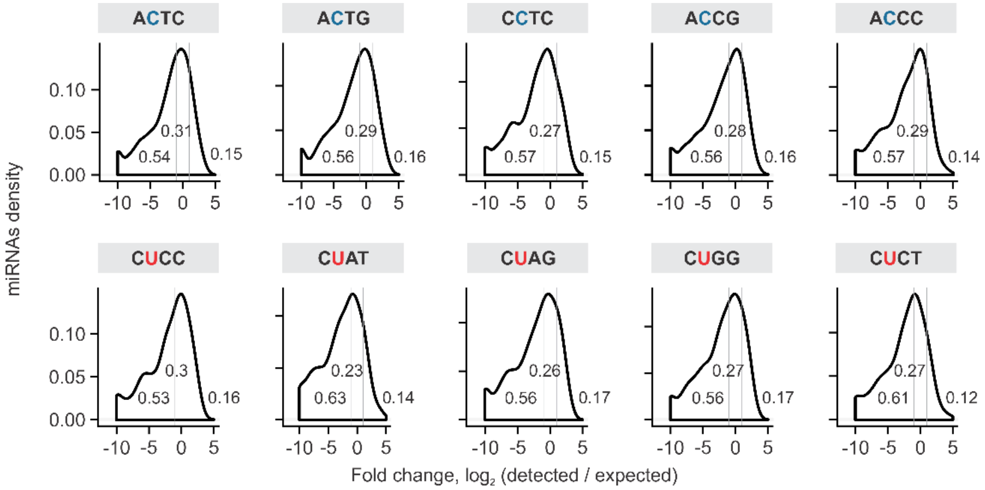

Fig. S8 Evaluation of the miRNA quantification accuracy using different 3' alkyne-adapters. CPMs were calculated using reference miRNAs cropped of 2 nt at the 3' end. log_2 fold-change of CPMs of identified miRNAs over the equimolar input was plotted. Values within two folds from the expected value (grey vertical lines) were considered unbiased according to. The data underlying the presented graphs are in Additional file 2.

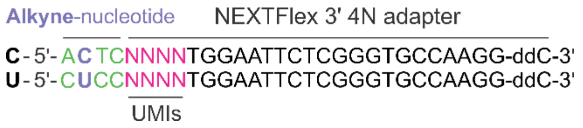

Fig. S9 Sequence of 3’ alkyne-adapters applied for *Lactobacillus casei* sRNAs sequencing.

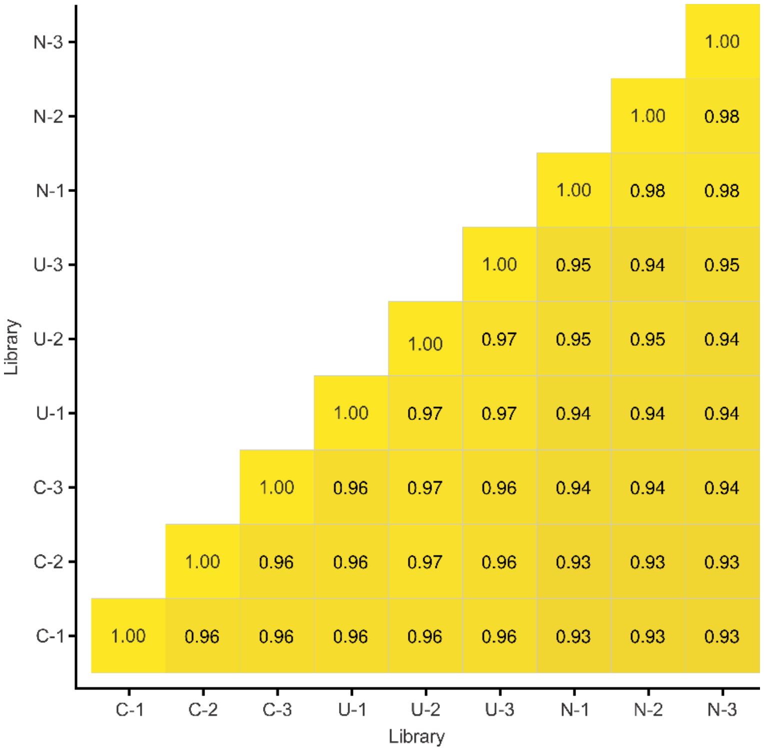

Fig. S10 Spearman correlation among biological replicates of the *L. casei* libraries. Bacterial genome was divided into non-overlapping 100 bp bins and the expression level in each bin was calculated using featureCounts program (excluding bins overlapping with rRNAs and tRNAs). Raw counts were normalized using TMM method and CPMs were used to calculate Spearman correlation coefficient

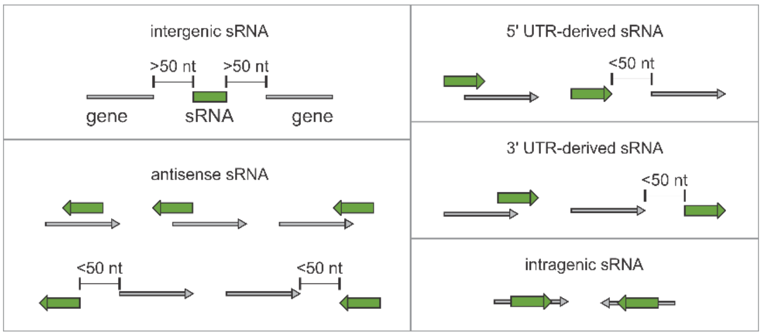

Fig. S11 Classification of sRNAs into groups based on their genomic context. Intergenic sRNAs are more than 50 nt away from annotated genes; antisense sRNAs are transcribed from opposite strand and fully or partially overlap with a gene including its 5' and 3' flanking regions of 50 nt; 5' UTR-derived sRNAs at least partially overlap with 5' end of a gene and its upstream region of 50 nt; 3' UTR-derived sRNAs at least partially overlap with 3' end of a gene and its downstream region of 50 nt; intragenic sRNAs are transcribed from the same strand and fully overlap with a cis-encoded gene. sRNAs that could be assigned to more than one group were termed “mixed” sRNAs.

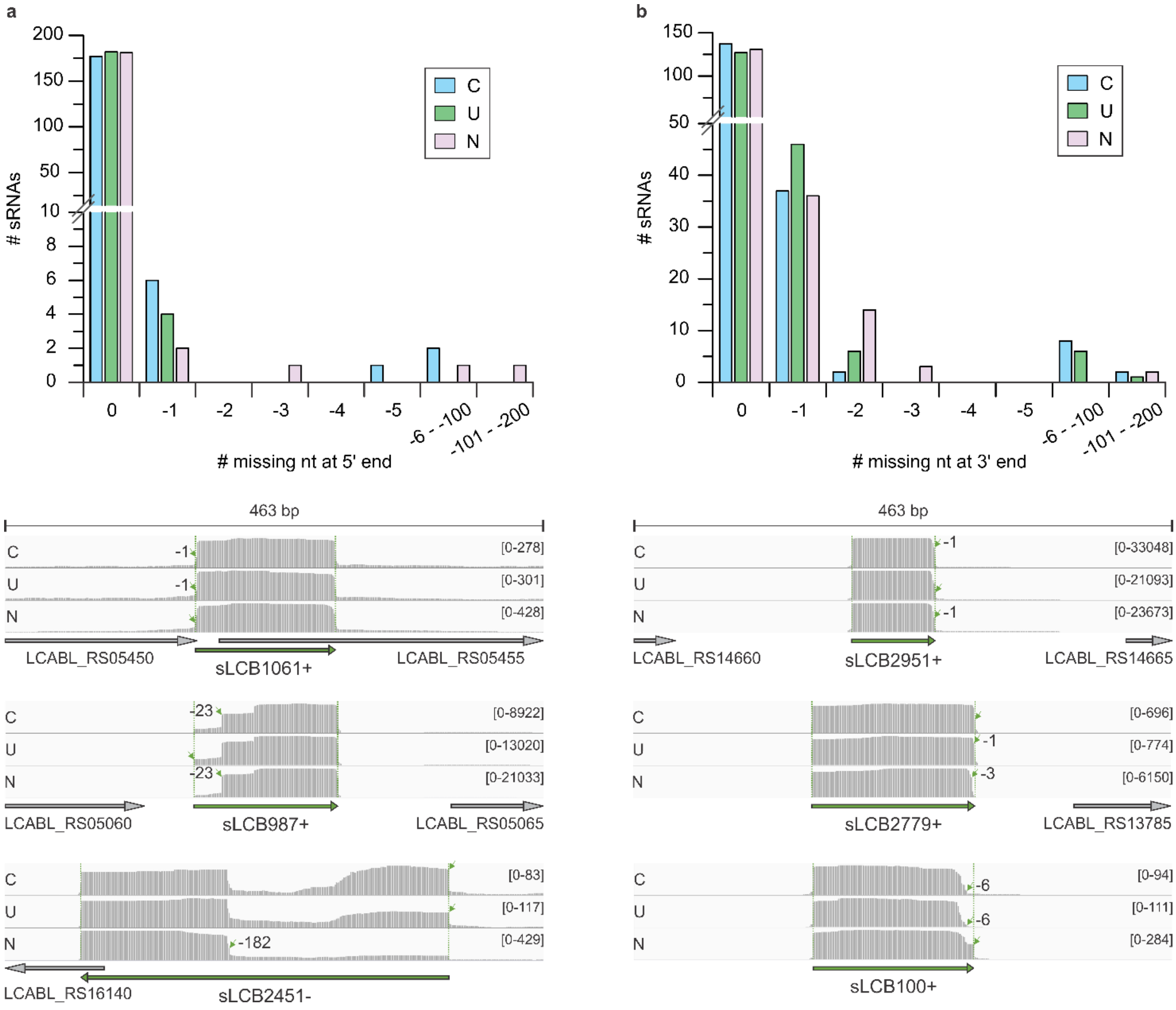

Fig. S12 The amount of predicted sRNAs with shorter 5' or 3' ends is similar among different libraries. **a** The majority of identified sRNAs have identical 5' ends in all three libraries. On the top, the column chart representing the portion of sRNAs with different number of missing nucleotides at the 5' end. Bottom, read coverage plots of few representative sRNAs with shorter 5' ends. Punctured green lines mark the first and last nucleotides of predicted sRNAs. Arrows point to the first nucleotide of predicted sRNA as observed in each library. **b** One or two missing nucleotides at the 3' end is similarly common for sRNAs identified in different libraries. On the top, the column chart representing the portion of sRNAs with different number of missing nucleotides at the 3' end. Bottom, read coverage plots of few representative sRNAs with shorter 3' ends. Arrows point to the last nucleotide of predicted sRNA as observed in each library. For both charts data were taken from Additional file 3: Table S2.

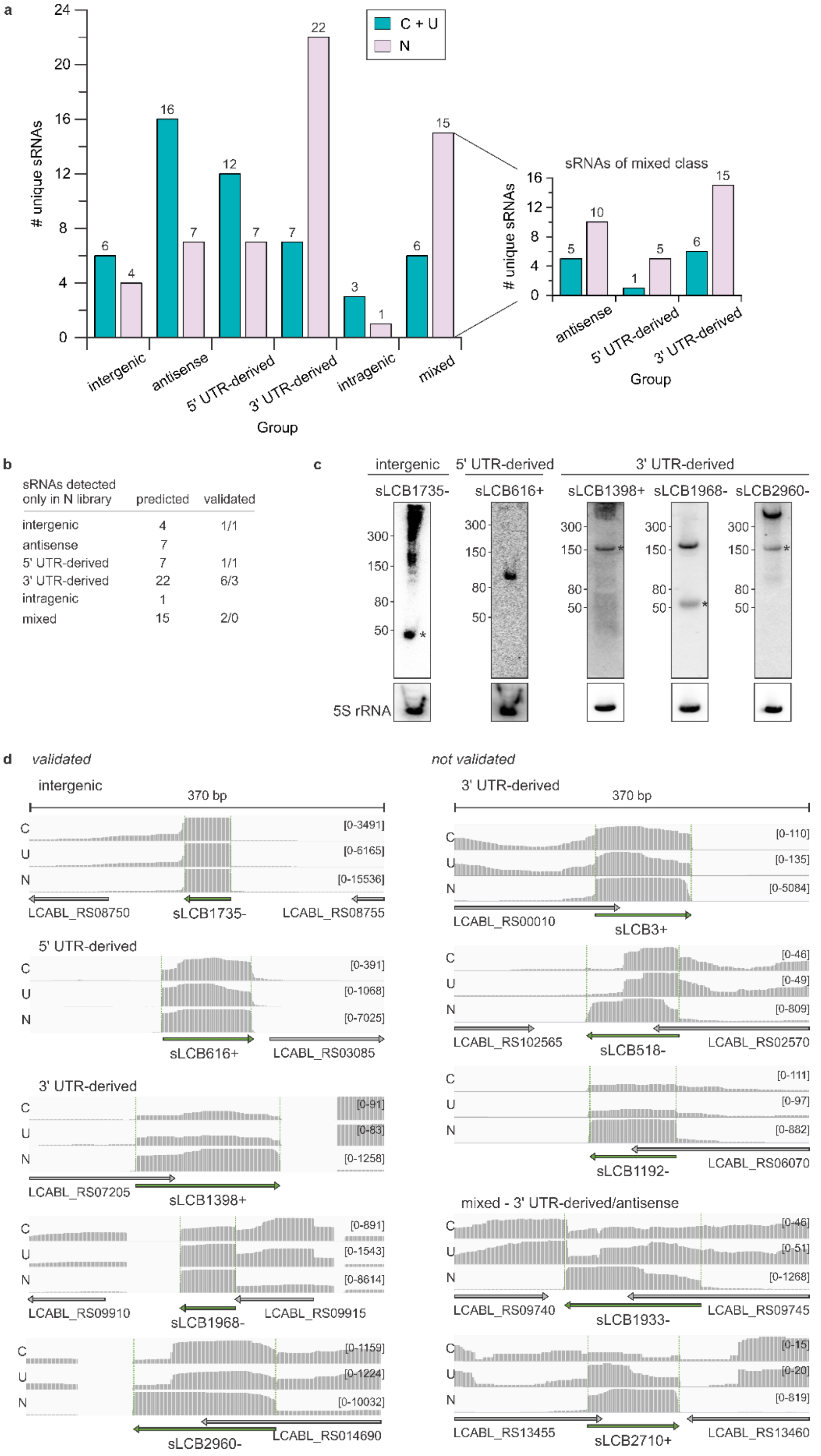

sRNAs detected only in N library	predicted	validated
intergenic	4	1/1
antisense	7	
5' UTR-derived	7	1/1
3' UTR-derived	22	6/3
intragenic	1	
mixed	15	2/0

Fig. S13 Putative 3' UTR-derived sRNAs tend to be incorrectly identified in the N library. **a** A considerable fraction of sRNAs detected only in the N library appears to be derived from 3' UTRs. **b** 3' UTR-derived sRNAs identified only in the N library are rarely validated. **c** Northern blot validation of sRNAs detected only in the N library. Asterixes mark sRNAs where multiple bands are visible. **d** Read coverage plots of sRNAs predicted only in the N library. For the convenience only sRNA strand-specific read coverage is shown.

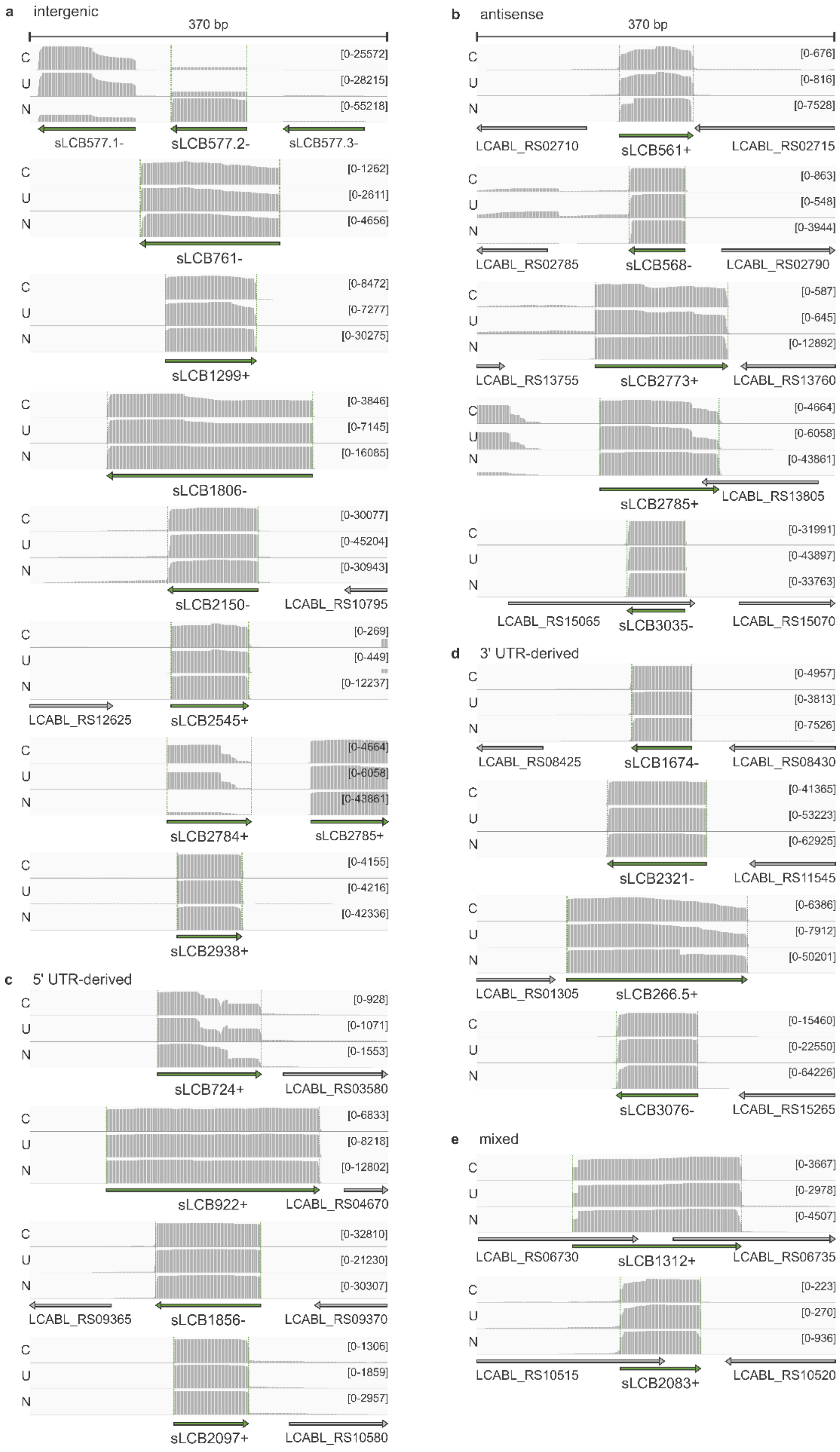

Fig. S14 Read coverage plots of experimentally verified sRNAs identified in all three libraries. sRNAs assigned to five different groups – intergenic **a**, antisense **b**, 5' UTR-derived **c**, 3' UTR-derived **d** and mixed **e** – were experimentally validated by Northern blot analysis. Punctured green lines mark the first and last nucleotides of predicted sRNA sequences. For the convenience only sRNA strand-specific read coverage is shown.

Table S1 The number of miRNA species captured in sequenced libraries. RNA sequences were truncated from the 3' end by the indicated number of nucleotides and searched for exact match against the libraries with no pre-set expression threshold. Only miRNAs exhibiting differences in their n-5 (where n denotes the length of miRNA) nucleotides at the 5' end were examined (893 in total).

Library → / 3' trimmed nt ↓	1C	2C	1U	2U
0	871	878	872	882
1	882	883	882	884
2	885	884	884	885
3	887	886	885	886
4	887	886	885	886
5	887	888	887	888
