# Supplementary material for: Methyltransferase-directed orthogonal tagging and sequencing of miRNAs and bacterial small RNAs
Source: BMC Biol. 2021 Jun 22;19:129. doi: 10.1186/s12915-021-01053-w (PMC8220740; doi:10.1186/s12915-021-01053-w)
Supplement: Supplementary file 4 — Additional file 4: Table S10. The list of RNA and DNA oligonucleotides used for labelling of double-stranded substrates with AtHEN1. Table S11. The list of L. casei BL23 5S rRNA specific probes used for rRNA depletion. Table S12. DNA oligonucleotides used for Northern blot analysis. [file 12915_2021_1053_MOESM4_ESM.pdf]

Table S10 The list of RNA and DNA oligonucleotides used for labelling of double-stranded substrates with AtHEN1.

**RNA**	**Sequence 5'-3'**
let7a	UGAGGUAGUAGGUUGUAUAGUU
let7a*	CUAUACAAUCUACUGUCUUUCC
miR-26a	UUCAAGUAAUCCAGGAUAGGCU
miR-26a*	CCUAUUCUUGGUUACUUGCACG
miR173	UUCGCUUGCAGAGAGAAAUCAC
miR173A	UUCGCUUGCAGAGAGAAAUCAA
miR173*	GAUUCUCUGUGUAAGCGAAAG
miR-210	CUGUGCGUGUGACAGCGGCUGA
miR-210*	AGCCCCUGCCCACCGCACACUG
**DNA**	**Sequence 5'-3'**
DNA7a	CTATACAACCTACTACCTCACC
DNA7a*	AAAGACAGTAGATTGTATAGAG
DNA26a	CCTATCCTGGATTACTTGAACG
DNA26a*	TGCAAGTAACCAAGAATAGGCT
DNA173	GATTTCTCTCTGCAAGCGAAAG
DNA173*	TTCGCTTACACAGAGAATCAC
DNA210	AGCCGCTGTCACACGCACAGTG
DNA210*	GTGTGCGGTGGGCAGGGGCTGA

Table S11 The list of *L. casei* BL23 5S rRNA specific probes used for rRNA depletion. +T – LNA nucleotide, G/A – G or A nucleotide at a particular position, Biotin-TEG – biotin attached to the 3' end of DNA oligonucleotide through the TEG spacer. LNA containing oligonucleotide was synthesised at *Exicon* and the rest were bought from *Metabion*.

**Probe**	**Sequence 5'-3'**
5S comp. 1	T+TCTGTGTTCGG/ACA+TGGGAACAGG+TGTATC-Biotin-TEG
5S *L. casei* comp. 2	CCACCAACTACTCTCGGCGTGAAGA-Biotin-TEG
5S *L. casei* comp. 3	AGCTTCCTACCCTCGCAGGCAGTTT-Biotin-TEG

Table S12 DNA oligonucleotides used for Northern blot analysis.

Probe	Sequence 5'-3'
BL23_5S	TGGCAGCTTCCTACCCTCGCAGGCAG
sLCB3+	CTAGAATCTTTTTTAATTGGCGCTCTTTGTTGC
sLCB518-	GTTGACCACATTAGCAACAAGCTAA
sLCB561+	ACCAAAGTGAACGGGTAGACGCCTAAC
sLCB568-	CTAGCACACTTAACGGCCAAAAATGAGGTTTCACAT
sLCB577.2-	GCTAGAAGATTAGGGTAGTACCGAGGAG
sLCB616+	CACACCCCTGAAAGAGAACACCTTGAGC
sLCB724+	GGTCTCGCCTAATTAAATAGTCACAATCC
sLCB761-	GTGGTGCCGACTTCTTTTTACTTTGG
sLCB922+	GGCTACTAATTTCACCCCAGCAACTTC
sLCB1192-	CGGATCAGTTATTGTCGGTAGTCTTCTT
sLCB1299+	GCTTCTACACCAGTCTTCCAGACCGTGTCG
sLCB1312+	AAACCACAGCGTTAGGCGATTAGGA
sLCB1398+	CATGTCTCCCAGAACCATTCCTTGTT
sLCB1674-	TGAAATGCGACTTTTTGATAGTCTATCGGCATAA
sLCB1735-	CATCGCTGCTCACTCACTGCCAGAACCT
sLCB1806-	GTTGAGTTGGATTCGATATGACATCG
sLCB1856-	CCACAAAGACCCCGCTGTTCATTAAATAC
sLCB1933-	CTATCAGCCGAATTAAGAAAATTAATGCA
sLCB1968-	CCATGCGTTTACGTCTTCTATCGAA
sLCB2083+	TTTGTTTGCCTATTTTTGACTACGATGGCG
sLCB2097+	GCCTTATTACGGAGTTTTCCCACTGCGACCC
sLCB2150-	CGAACTTATTCACTTGTTGGCACGTTTGC
sLCB2321-	GTACAAGTCGTGTCTTTCTGGTTTTGCC
sLCB2545+	CCTTCTAAATACGCATAAGGTTTG
sLCB266.5+	AAGTTGACTTGGATAGGATCTCGCTGG
sLCB2710+	CGTTTTTTATGTGGTTGTCAAATGATTCG
sLCB2773+	GGAGGAGAAAAATGAAAATTAGGTTGGGGTTTAGG
sLCB2784+	GTTTTGGTGACGGTCTTTTTAGGGAGTGCTG
sLCB2785+	GATTGCTTAAACGAATGTTGACCGTCTTC
sLCB2938+	GTACAGGGGAAACGGCTATTGTGATGTGACC

sLCB2960-	GCTAGGCAAATAATTCGTAGATCAATC
sLCB3035-	ACCTCAAGGCGAGAAGAAGACAAG
sLCB3076-	CTGGATAAAGCACTTTGACTACTTCATCG
